# Network pharmacology, molecular docking, and experimental validation to explore the potential mechanism of Long Mu Qing Xin mixture for the treatment of attention deficit hyperactivity disorder

**DOI:** 10.3389/fphar.2023.1144907

**Published:** 2023-03-17

**Authors:** Xuejun Li, Zhen Xiao, Wenyan Pu, Zhiyan Jiang, Shumin Wang, Yixing Zhang

**Affiliations:** ^1^ Pediatrics, Longhua Hospital Affiliated to Shanghai University of Traditional Chinese Medicine, Shanghai, China; ^2^ Longhua Clinical Medical College, Shanghai University of Traditional Chinese Medicine, Shanghai, China

**Keywords:** attention deficit hyperactivity disorder, Long Mu Qing Xin Mixture, network pharmacology, molecular docking, dopamine, cAMP/PKA signaling pathway, spontaneously hypertensive rats

## Abstract

**Background:** Long Mu Qing Xin Mixture (LMQXM) has shown potentially positive effects in alleviating attention deficit hyperactivity disorder (ADHD); however, the action mechanism is still not fully understood. This study aimed to predict the potential mechanism of LMQXM for ADHD using network pharmacology and molecular docking, which were then validated using animal experiments.

**Methods:** Network pharmacology and molecular docking techniques were used to predict the core targets and potential pathways of LMQXMQ for ADHD, and KEGG pathway enrichment analysis revealed the potential significance of dopamine (DA) and cyclic adenosine monophosphate (cAMP) signaling pathways. To verify the hypothesis, we conducted an animal experiment. In the animal experiment, the young spontaneously hypertensive rats (SHRs) were randomly divided into the model group (SHR), the methylphenidate hydrochloride group (MPH, 4.22 mg/kg), and 3 LMQXM groups (low-dose (LD) group, 5.28 ml/kg; medium-dose (MD) group, 10.56 ml/kg; and high-dose (HD) group, 21.12 ml/kg), and administered by gavage for 4 weeks; the WKY rats were set as the control group. The open field test and Morris water maze test were used to evaluate the behavioral performance of rats, high performance liquid chromatography mass spectrometry (LC-MS) was used to analyze DA levels in the prefrontal cortex (PFC) and striatum of rats, ELISA was used to detect cAMP concentrations in the PFC and striatum, and immunohistochemistry and qPCR were used to analyze positive cell expression and mRNA expression for indicators related to DA and cAMP pathways.

**Results:** The results showed that beta-sitosterol, stigmasterol, rhynchophylline, baicalein, and formononetin might be key components of LMQXM for ADHD and that these components bind well to the core targets, DA receptors (DRD1 and DRD2). Furthermore, LMQXM might act through the DA and cAMP signaling pathways. In the animal experiment, we found that MPH and LMQXM-MD controlled hyperactivity and improved learning and memory in SHRs, while LMQXM-HD only controlled hyperactivity in SHRs; meanwhile, MPH and LMQXM-MD upregulated DA and cAMP levels, mean optical density (MOD) of cAMP, and MOD and mRNA expression of DRD1 and PKA in the prefrontal cortex (PFC) and striatum of SHRs, while LMQXM-LD and LMQXM-HD upregulated DA and cAMP levels in the striatum, MOD of cAMP in the PFC, and mRNA expression of PKA in the PFC. However, we did not find a significant regulatory effect of LMQXM on DRD2.

**Conclusion:** To sum up, this study demonstrated that LMQXM may increase DA levels mainly by activating the cAMP/PKA signaling pathway through DRD1, thereby controlling the behavioral disorders of SHRs, which is most effective at moderate doses, and this may be a key mechanism for LMQXM in the treatment of ADHD.

## 1 Introduction

Attention deficit hyperactivity disorder (ADHD) is a common, early-onset, persistent neurodevelopmental disorder in children (Banaschewski et al., 2017), with inattention, hyperactivity, and impulsivity as core symptoms, often accompanied by learning and memory deficits. The global prevalence of ADHD is about 5.29% (Posner et al., 2020), with boys having a higher incidence (Mahone and Denckla, 2017). In recent years, the prevalence rate of ADHD has been increasing (Carbray, 2018), and about 50% of patients have symptoms that persist into adulthood. In addition, these patients often face the risk of academic failure, accidental injury, criminal behavior, and even suicide (Faraone and Larsson, 2019), which brings a heavy psychological and economic burden to individuals, families, and society. Dopamine (DA) defects or dysregulated DA transmission is considered to be the core pathogenesis of ADHD (Drechsler et al., 2020); however, the specific pathogenesis has not yet been elucidated, and the prefrontal cortex (PFC) and striatum, as key regions for DA neurotransmission, have become a mainstream direction for studying attention and learning and memory deficits in ADHD (Sharma and Couture, 2013; Ruocco et al., 2014).

As methylphenidate (MPH) can increase the availability of extracellular DA in the synaptic gap by blocking dopamine transporter protein (DAT) (Faraone, 2018; Rostami Kandroodi et al., 2021), it has been used as a first-line drug for the treatment of ADHD. However, some scholars have suggested that children should be at least 6 years old when administered MPH because the long-term use of this drug could lead to addiction (Verghese and Abdijadid, 2022). Furthermore, MPH has a risk of inducing abnormal behaviors such as anxiety, depression, sleep disorders, and even suicide (Ekhart et al., 2020). Considering these adverse factors, the main purpose of medical research is to explore safer and more effective treatment strategies based on ADHD neuropathological and physiological mechanisms.

Long Mu Qing Xin Mixture (LMQXM) is an empirical Chinese medicine formula for ADHD in children, consisting of radix astragail, radix angelicae sinensis, ramulus uncariae cum uncis, fructus jujubae, radix paeoniae alba, fructus schisandrae, radix scutellariae, cortex phellodendri, calcined dragon bone, calcined oyster shell, conch margartifera usta, magnetitum, mix-fried licorice, light wheat, and caulis polygoni multiflori, which can tonify and replenish the heart and spleen, nourish the heart and tranquilize the mind, and calm the liver and subdue yang. In a previous study, we used LMQXM to perform a small clinical randomized control study on 144 children with ADHD and found that LMQXM significantly improved abnormal behavior such as hyperactivity, inattention, impulsivity, and learning difficulties in patients, and the disease efficacy control rate was significantly better in the trial group than in the pediatric intelligence syrup control group (86.67% vs 50%, *p* < 0.05) (Chen and Xiao, 2016; Zhang and Jiang, 2016), which showed that LMQXM may be a potential new therapy to alleviate the symptoms of ADHD, but its targets of action and molecular mechanisms remain unclear. In addition, we also found that LMQXM improved learning and memory in ADHD model mice (Sun et al., 1991), so we hypothesized that LMQXM may exert therapeutic effects by acting on certain pathological processes in ADHD.

Network pharmacology can reflect the multi-component and multi-target characteristics of traditional Chinese medicine and has been widely used in traditional Chinese medicine research and advanced drug discovery (Xu et al., 2021). This study aimed to identify the key targets and pathways involved in LMQXM alleviating ADHD through network pharmacology and molecular docking and to conduct an animal experiment to verify the effect of LMQXM on behavioral performance in spontaneously hypertensive rats (SHRs). SHR is derived from an inbred strain of the Wistar-Kyoto (WKY) rat and exhibits similar etiological, pathological, biochemical, and symptomatological features to ADHD (Song et al., 2021). MPH increased attention and memory capacity and decreased impulsivity in SHRs (Leffa et al., 2019), and altered the expression of genes associated with impulse characteristics, such as nuclear receptor 4a2 (NR4A2), B-cell translocation gene 2 (Btg2), and Homer2 (Dela Peña et al., 2017), indicating that SHRs showed a positive response to neurostimulants and can better predict ADHD-related pathological mechanisms. SHR is considered a commonly used and well-studied animal model of ADHD because it exhibits behavioral disorders similar to ADHD in terms of hyperactivity, impulsivity, and learning disabilities, predicts molecular genetics and neuropathological mechanisms associated with ADHD, and responds to first-line ADHD therapeutic agents (Rahi and Kumar, 2021). Since ADHD is more common in boys than in girls, and male rats are superior to females in terms of surface validity (hyperactive/impulsive behavior), male SHRs are more suitable as an animal model of ADHD (Bayless et al., 2015; Kozłowska et al., 2019). This study attempted to use male SHRs as the ADHD animal model and WKY rats as the normal control to investigate the potential mechanisms of LMQXM for ADHD and explore the effects of LMQXM on behavioral performance and related factors in SHRs. This is the first study of the behavioral effects of LMQXM on SHRs and the potential molecular mechanisms for the treatment of ADHD, which will provide a research basis and new directions for experimental pharmacological studies in ADHD.

## 2 Materials and methods

### 2.1 Active ingredient screening and target prediction of LMQXM

Traditional Chinese Medicine Systems Pharmacology Database and Analysis Platform (TCMSP) (Zhang et al., 2019) (https://tcmspw.com/tcmsp.php) was used to screen LMQXM active ingredients and targets. ADME reference standards were as follows: oral bioavailability (OB) ≥ 30%, blood-brain barrier (BBB) permeability ≥ −0.3, drug-like properties (DL)≥0.18 (Yi et al., 2020). For ingredients not found in TCMSP, we used the ChemScr database (https://www.chemsrc.com/) to query the CAS number of the compound, after which we used the PubChem database (Kim et al., 2021) (https://pubchem.ncbi.nlm.nih.gov/) to download the 2D molecular structure of the compound and uploaded them to SwissADME (http://www.swissadme.ch/), SwissTargetPrediction database (Daina et al., 2019) (http://www.swisstargetprediction.ch), and then based on standards of GI absorption (GIA) as “High”, BBB permeant as “YES”, DL had at least two meeting the criteria of “YES” and ‘*Homo sapiens*, Probability≥0.12′to screen active ingredients and predict the corresponding targets (Han et al., 2020). Duplicate targets of the drugs were removed, and the final targets were imported into the Uniprot database (Yi et al., 2020) (http://www.uniprot.org/) for normalization.

### 2.2 Construction of Chinese medicine-active ingredient-target network

Cytoscape 3.7.2 (Otasek et al., 2019) was used to construct the Chinese medicine-active ingredient-target network to elucidate the relationship between Chinese medicine, active ingredients, and targets in LMQXM. Among them, “node” represented Chinese medicine, active ingredients, and targets, “edge” represented the inter-action relationship between each node of Chinese medicine, ingredients, and targets, and “degree” indicated the number of edges connected to them. The number of node degree values was positively correlated with the importance of nodes in the network.

### 2.3 Screening of the intersection targets of LMQXM and ADHD

DrugBank database (Wishart et al., 2006.) (https://go.drugbank.com/), GeneCards database (Rappaport et al., 2017) (https://www.genecards.org/), Therapeutic Target Database (TTD) (Wang et al., 2020a) (http://db.idrblab.net/ttd/), DisGeNET database (Piñeroet al., 2020) (https://www.disgenet.org/) were used to screen ADHD-related targets, and the Uniprot database was used to normalize disease targets. LMQXM-related targets and ADHD-related targets were imported into Venny2.1.0 (https://bioinfogp.cnb.csic.es/tools/venny/index.html) to create Venn diagrams and screen intersection targets.

### 2.4 PPI network construction and core targets screening of LMQXM for ADHD

The intersection targets were uploaded to the STRING database (Szklarczyk et al., 2015) (https://cn.string-db.org/) to establish a protein-protein interaction (PPI) network for the treatment of ADHD with LMQXM. The “TSV” file of the PPI network was imported into Cytoscape 3.7.2 software for visualization processing. The Network Analyzer function was used to analyze the topological attributes of the PPI network and calculate the degree values, BC, and CC of each node. Targets above the median valuesof degree values, BC, and CC were selected as the core targets of LMQXM for ADHD (Li et al., 2022).

### 2.5 GO and KEGG pathway enrichment analysis

The core targets of LMQXM for ADHD were submitted to the Metascape platform (Zhou et al., 2019) for gene ontology (GO) and Kyoto encyclopedia of genes and genomes (KEGG) pathway enrichment analysis. GO enrichment analysis (Chen et al., 2017) is a bioinformatics program that covers biological processes, molecular functions, and cellular components. Biological processes involve the biological goals facilitated by genes or gene products; molecular functions are considered biochemical activities; cellular components refer to the parts of the cell where the gene product is active. KEGG (Chen et al., 2017) is a com-prehensive knowledge base for functional interpretation and practical application of genomic information. Based on the species set as “*H. sapiens*”, *p* < 0.01, the core targets were subjected to GO and KEGG enrichment analysis. The results were visualized using the online platform for microbial information (http://www.bioinformatics.com.cn/).

### 2.6 Molecular docking

The core active ingredients were selected as ligands, and the 3D molecular structures (SDF format) of the compounds were downloaded from the PubChem database and converted to mol two format using OpenBabel 3.1.1. The core targets were used as the receptors, and the 3D structures (PDB format) of the targets were downloaded from the RCSB Protein Data Bank (PDB) database (Rose et al., 2020) (https://www.rcsb.org/). The selection of the target protein was based on the following principles (Yang et al., 2016): 1) human protein; 2) high resolution; 3) the original ligand was preferred. PyMOL 2.4.1 (Mooers, 2020) was used to remove water molecules and original ligands from the core target protein. AutoDock 4.2.6 software (Goodsell et al., 2021) was used to hydrogenate the core target and convert it to pdbqt format, at the same time, the small molecule compound rotation bond was set and saved in pdbqt format. Molecular docking of core target proteins and small molecule compounds was performed using semi-flexible molecular docking, and the results were visualized with PyMOL software.

### 2.7 Animal experiment verification

The flow chart of the animal experimental study can be found in Supplementary Figure S1.

#### 2.7.1 Drug preparation

LMQXM (batch number: 2206001, HYZZ: Z05170218) was purchased from Longhua Hospital affiliated with the Shanghai University of Traditional Chinese Medicine. Preparation of medicinal solution was performed as follows: DLG, DML, ZZM, and CS were first decocted twice in distilled water of 5 times volume for 2 h each time, and then mixed with HQ, DG, GT, ZGC, DZ, FXM, BS, WWZ, YJT, HQIN, and HB. After being decocted two times, the filtrate was combined and concentrated into 4.17 g/ml crude herbal medicine by a rotary evaporator.

Methylphenidate hydrochloride sustained-release tablets (MPH, batch number: 1KE744) were provided by Xi’an Janssen Pharmaceutical Co, Ltd. A suspension was prepared with a concentration of 0.21 mg/ml with normal saline.

#### 2.7.2 Component identification of LMQXM

One ml of LMQXM sample solution was diluted 2 times with 20% methanol, mixed well, and centrifuged at 12000 r/min for 5 min, after which the supernatant was extracted. The chemical composition of LMQXM was characterized by ultra-performance liquid chromatography-tandem quadrupole time-of-flight mass spectrometry (UPLC-Q-TOF-MS) in positive and negative ion modes. A total of 40 components were identified, including astragaloside, formononetin, baicalin, baicalein, rhynchophylline, isorhynchophylline, etc., which may be the material basis of LMQXM for the treatment of ADHD. The results of component identification and mass spectra are shown in Supplementary Table S1, Supplementary Figure S2).

#### 2.7.3 Experimental animal

SPF grade 4-week-old male SHRs (*n* = 30) and WKY rats (*n* = 6) were purchased from Beijing Weitong Lihua Experimental Animal Technology Co., Ltd (Beijing, China, SCXK (Jing) 2021-0006) and raised in the barrier environment of the Experimental Animal Center of Shanghai University of Traditional Chinese Medicine. All the animals were housed in an environment with a temperature of 23ºC ± 2°C, relative humidity of 55% ± 5%, and a light/dark cycle of 12/12 h, and had free access to water and food. All rats received adaptive feeding for 1 week before drug administration. All animal studies (including the mice euthanasia procedure) were done in compliance with the regulations and guidelines of Shanghai University of Traditional Chinese Medicine institutional animal care and conducted according to the AAALAC and the IACUC guidelines (No. PZSHUTCM220711003).

#### 2.7.4 Drug intervention

After 1 week of adaptive feeding, the rats will begin to receive treatment (5 weeks of age). The SHRs were divided into five groups according to the random number table: the model group (SHR), the methylphenidate hydrochloride group (MPH, 4.22 mg/kg), the LMQXM low dose group (LMQXM-LD, 5.28 ml/kg), the LMQXM medium dose group (LMQXM-MD, 10.56 ml/kg), and the LMQXM high dose group (LMQXM-HD, 21.12 ml/kg); WKY rats were used as a normal control group. The sample size of animals was determined by the resource equation method (Festing and Altman, 2002). The control and model groups were given normal saline by gavage, and the MPH and LMQXM groups were given corresponding drugs by gavage twice a day (8:00-9:00 a.m.; 14:00-15:00 p.m.) for 4 weeks, with a volume of 1 ml/100 g. Doses administered to rats were converted in parallel based on the body surface area of 9-year-old (26 kg body weight) children (0.991 m^2^, Stevenson formula) and the body surface area of 4-week-old (60 g body mass) SHR pups (0.013947 m^2^, Meeh-Rubner formula). Rats were weighed daily before gavage to determine the volume of the drug.

#### 2.7.5 Behavioral tests

According to the previous behavioral testing methods, the open field test (OFT) was conducted at 0 and 4 weeks of treatment (at 5 and 9 weeks of age in rats) to evaluate the locomotor activity of rats, and the Morris Water Maze (MWM) test was performed after 3 weeks of treatment (at 8–9 weeks of age in rats) to evaluate the learning and memory abilities of the animals (Ding et al., 2017).

##### 2.7.5.1 Open field test (OFT)

The experimental equipment consisted of a black open field test box (100 cm × 100 cm × 48 cm), a camera, and a computer. The rats were gently placed in the central area of the test box (50 cm × A 50 cm area in the center of the test box), and a camera tracked the movement of each animal. Fiji Is Just ImageJ (Fiji) software was used to analyze the movement distance, speed, and time spent in the central area of the rats. The activity of each rat was observed for 5 min (Song et al., 2021; de Sousa Macedo et al., 2022). After the test, 75% alcohol was used to remove the odor.

##### 2.7.5.2 Morris Water Maze (MWM) test

The MWM device consisted of a black round pool (150 cm in diameter and 50 cm in depth), a camera, and a computer tracking system (Ethovision XT). The pool was filled with water (23°C ± 2°C) and dyed with black ink to distinguish a rat inside the box more easily. The system software divided the water maze into four quadrants. The black round platform (12 cm in diameter) was located in the quadrant Ⅱand immersed about 1 cm below the water surface. White geometric figures of approximately 10 cm in size and differing shapes were set up on the inner wall of the pool above the horizontal plane as spatial references to help rats navigate spatially. The tests were conducted for 6 days. From day 1–5, rats were trained once a day and the time searching the escape platform was recorded; the maximum swim time was set to 60 s. If the mouse located the platform before 60 s had passed, it was immediately removed from the pool (this was recorded as escape latency). If the platform was not located after 60 s, the mouse was gently guided to the platform and allowed to re-orient to the distal visual cues for an additional 10 s before being removed from the pool. The sixth day was the probe trial, during which the platform was removed and the annulus visits and time spent in the target quadrant (s) within the 60 s were recorded by the Ethovision XT system.

#### 2.7.6 Collection of prefrontal cortex and striatum samples

After the behavioral test, the rats were fasted for 12 h and anesthetized with Zoletil ^®^ (50 mg/kg). After decapitation of rats the PFC and striatum were quickly separated on the ice surface with a glass minute needle. The samples were quickly frozen in liquid nitrogen and stored at −80°C.

#### 2.7.7 High performance liquid chromatography mass spectrometry (LC-MS)

UltiMate 3000 RS chromatograph and TSQ Quantum triple quadrupole mass spectrometer were used to analyze DA levels in the PFC and striatum. Chromatographic conditions were as follows: the chromatographic column was Welch Ultimate XB-C8 (150 mm × 4.6 mm, 5 μm), the mobile phase consisted of pump A (0.1% formic acid aqueous solution) and pump B (0.1% formic acid acetonitrile), the gradient of the mobile phase was 0 0min, 90% A-10% B; 2 min, 5%A-95%B; 4 min, 5%A-95%B; 4 min, 90%A-10%B; 5 min, and 90%A-10%B. The flow rate was 0.8 ml/min, the column temperature was 40°C, and the injection volume was 2 μL. Mass spectrometry conditions were as follows: an ion source, electrospray ionization source; scan mode, positive and negative ion switching scan; detection mode, selected reaction monitoring; electrospray voltage (Spary Voltage) (+) 4000 V capillary temperature (Source Temperature), 350°C; atomization temperature (Vaporizer Temperature): 500°C; collision gas, high-purity argon (purity ≥99.999%); sheath gas (Sheath Gas Pressure), nitrogen (purity ≥99.999%), 65Arb; auxiliary gas (Aux Gas Pressure), nitrogen (purity ≥99.999%), 20Arb; data acquisition time, 5min.

#### 2.7.8 Enzyme-linked immunosorbent assay (ELISA)

The Rat ELISA kit (item number ER9351M) was purchased from BIOTHEC WELL (Shanghai, China), and cAMP concentration levels in the PFC and striatum were measured according to the protocol provided by the manufacturer.

#### 2.7.9 Immunohistochemistry

Three rats were randomly selected from each group, and the PFC and striatum were fixed with 4% paraformaldehyde, then embedded in paraffin and cut into 3-4 μm thick sections. After antigen repair, samples were treated with 3% H_2_O_2_ (to block endogenous peroxidase activity) and serum/BSA, and then incubated with primary antibody (see Table 1 for corresponding antibody information) at 4°C overnight. Samples were then washed and incubated with a secondary antibody (HRP-labeled goat anti-rabbit IgG, 1:200 dilution, room temperature, 50 min) for 2 h at room temperature. Then DAB color development (positive expression is brownish yellow), hematoxylin re-staining of cell nuclei (3 min), and dehydration and sealing of the sections were performed. The sections were scanned under a microscope to observe the expression of dopamine D1 and D2 receptor (DRD1 and DRD2), cyclic adenosine monophosphate (cAMP), and protein kinase A (PKA). Image-Pro Plus 6.0 software was used to analyze related indicators’ mean optical density value (MOD).

**TABLE 1:** Antibody information.

Name	Item no.	Manufacturers	Genus	Dilution ratio
cAMP	A15653	abclonal	rab	1:200
PKA	ER1706-65	huabio	rab	1:200
DRD2	55084-1-AP	Proteintech	rab	1:500
DRD1	ET1703-45	huabio	rab	1:200

#### 2.7.10 Fluorescent quantitative PCR analysis

The primer sequences are shown in Table 2. Total mRNA was extracted from the prefrontal and striatum using Trizol, and RNA concentration and purity were detected at 260 and 280 nm by ultra-micro spectrophotometer (Piñero et al., 2020). ®RT first strand cDNA synthesis kit was used to reverse transcribe RNA to cDNA. The following conditions were applied: 25°C for 5 min, 42°C for 30 min, and 85°C for 5 s. PCR amplification conditions were: 95°C for the 30 s, 95°C for 15s, 60°C for 30 s, 40 cycles. Fluorescence quantitative PCR instrument (Bio-rad) output the experimental results, while 2^-△△Ct^ algorithm was used to analyze the relative expression of PFC and striatal PKA, DRD1, and DRD2 mRNA.

**TABLE 2:** qPCR primer sequences.

Gene name	—	Primer sequences
PKA (189 bp)	Upstream	CCG​AAC​TTG​GAC​CTT​GTG​TGG
	Downstream	CGC​ACC​TTC​CCA​GAG​ACG​ATT
DRD1 (125 bp)	Upstream	GTG​CTC​TAC​GGC​GTC​CAT​TC
	Downstream	CTA​CGC​TAA​TCA​GGA​TGA​AGG​CT
DRD2 (290 bp)	Upstream	AAC​CCT​GAC​AGT​CCT​GCC​AAA
	Downstream	CAT​GTG​AAG​GCG​CTG​TAG​AGG​A
Rat-GAPDH (138 bp)	Upstream	CTG​GAG​AAA​CCT​GCC​AAG​TAT​G
	Downstream	GGT​GGA​AGA​ATG​GGA​GTT​GCT

### 2.8 Statistical analysis

Data were analyzed using SPSS 25.0 software, and the measurement data were expressed as mean ± standard deviation. One-way analysis of variance (ANOVA) was used for comparison between groups, and LSD and Dunet’s method were used for multiple comparisons. A two-way ANOVA was used to analyze data sets involving treatments and time points. *p* < 0.05 indicated statistically significant differences. Graphs were plotted using GraphPad Prism 8.0 software.

## 3 Results

### 3.1 Active ingredient screening and target prediction of LMQXM

The number of active ingredients and targets of LMQXM were shown in Table 3. We eliminated duplicate compounds and targets and filtered compounds without target information, resulting in 177 active ingredients and 355 corresponding targets. The information on related active ingredients was shown in Supplementary Table S2. The shared ingredients of LMQXM such as mairin, beta-sitosterol, stigmasterol, rhynchophylline, hirsutine, berberine, fumarine coptisine, and palmatine may be the key ingredients through which LMQXM exerts therapeutic effect (Table 4).

**TABLE 3:** The number of active ingredients and related targets of LMQXM.

Chinese medicine	Chinese name	Active ingredient	Target
Radix Astragail	Huangqi (HQ)	12	67
Radix Angelicae Sinensis	Danggui (DG)	2	50
Ramulus Uncariae cum Uncis	Gouteng (GT)	27	76
Fructus jujubae	Dazao (DZ)	18	84
Radix Paeoniae Alba	Baishao (BS)	3	36
Fructus Schisandrae	Wuweizi (WWZ)	8	16
Radix Scutellariae	Huangqin (HQIN)	27	116
Cortex Phellodendri	Huangbai (HB)	27	87
Calcined Os Draconis	Duan-Longgu (DLG)	32	107
Calcined Concha Ostreae	Duan-Muli (DML)	6	34
Conch Margartifera Usta	Zhenzhumu (ZZM)	18	20
Magnetitum	Cishi (CS)	8	48
Honey-fijed Licorice	Zhigancao (ZGC)	18	40
Tritici Levis	Fuxiaomai (FXM)	49	69
Caulis Polygoni Multiflori	Shouwuteng (SWT)	22	138

**TABLE 4:** Detailed information of shared active ingredients derived from LMQXM.

Mol ID/CAS	Number	Mol name	Mol structure	Mol formula	OB/GIA	BBB	DL	Related Chinese medicine
MOL000211	A1	Mairin	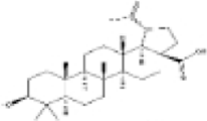	C_30_H_48_O_3_	55.38	0.22	0.78	HQ, DZ, BS
MOL000358	B1	Beta-sitosterol	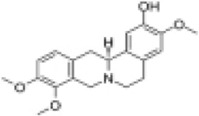	C_29_H_50_O	36.91	0.99	0.75	DG, GT, DZ, BS, HQIN, HB
MOL000449	B2	Stigmasterol	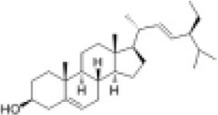	C_29_H_48_O	43.83	1	0.76	DG, DZ, HQIN, HB
MOL000359	C1	Sitosterol	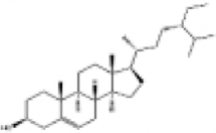	C_29_H_50_O	36.91	0.87	0.75	GT, BS, HQIN
MOL008469	C2	Rhynchophylline	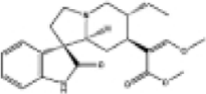	C_22_H_28_N_2_O_4_	41.82	0.38	0.57	GT, SWT
MOL008487	C3	Hirsutine	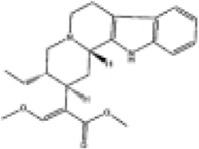	C_22_H_28_N_2_O_3_	34.44	0.78	0.43	GT, SWT
MOL001454	E1	Berberine	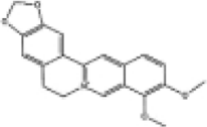	C_20_H_18_NO_4_^+^	36.86	0.57	0.78	DZ, HB
MOL000787	E2	Fumarine	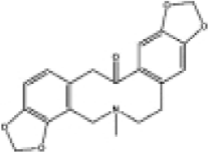	C_20_H_19_NO_5_	59.26	−0.13	0.83	DZ, HB
MOL001458	D2	Coptisine	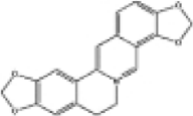	C_19_H_14_NO_4_^+^	30.67	0.32	0.86	HQIN, HB
MOL002897	D1	Epiberberine	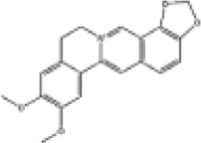	C_20_H_18_NO_4_^+^	43.09	0.4	0.78	HQIN, FXM
MOL000785	E3	Palmatine	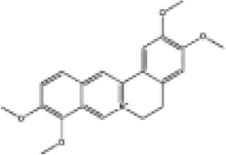	C_21_H_22_NO_4_^+^	64.6	0.37	0.65	HB, SWT

### 3.2 Construction of the Chinese medicine-active ingredient-target network

**FIGURE 1:**
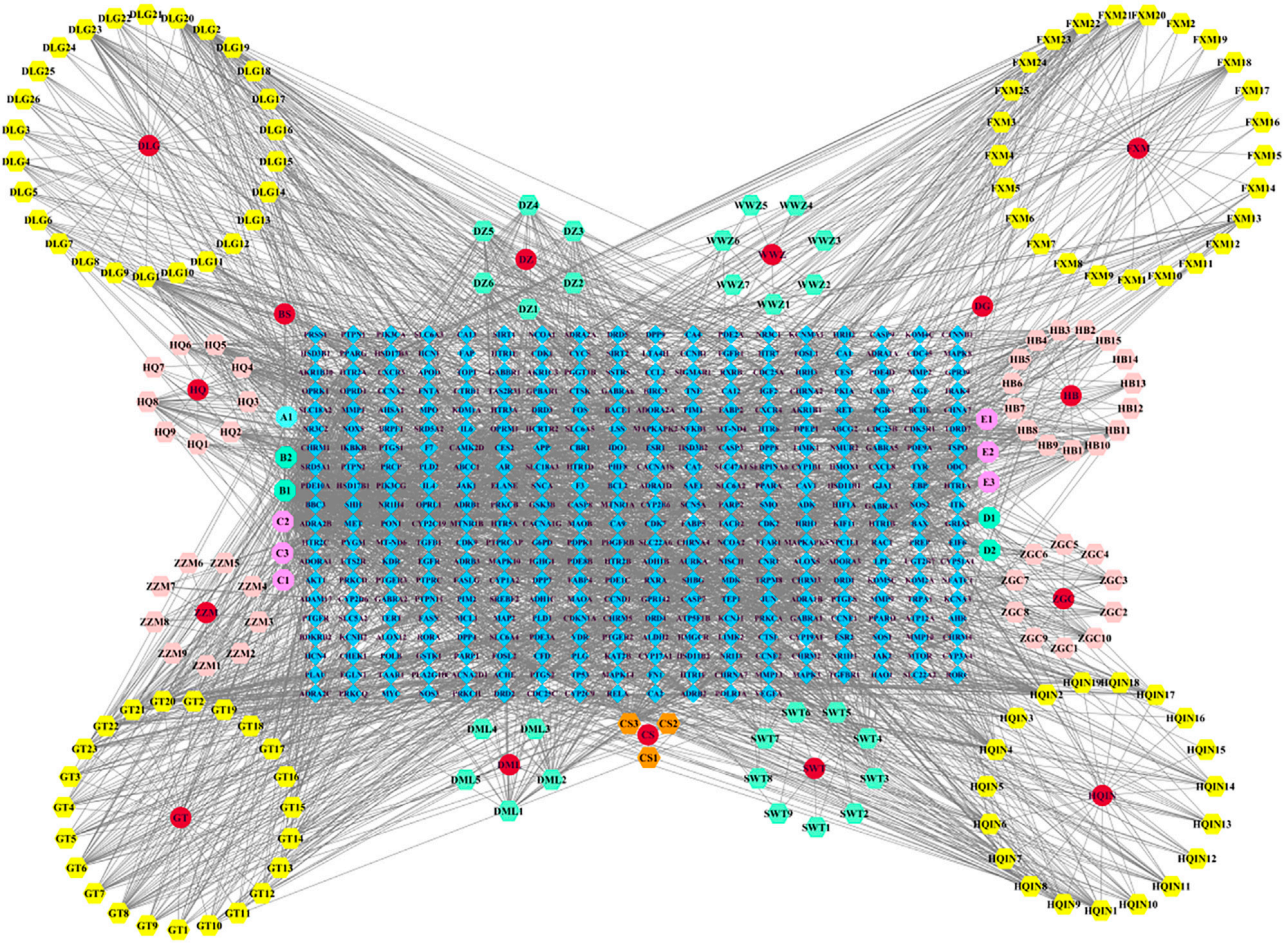
Chinese medicine-active ingredient-target network of LMQXM. The red circle nodes represent Chinese medicines; the regular hexagons represent the active ingredients; the regular octagons represent the common components between Chinese medicines; the blue diamond nodes represent the drug targets.

We imported the created “network” and ‘type’ files into Cytoscape 3.7.2 to construct a Chinese medicine-active ingredient-target network (Figure 1). The results revealed that the network contained 547 nodes (15 Chinese medicine nodes, 177 ingredient nodes, and 355 target nodes) and 2,729 edges. The “Analyze Network” function was used to calculate the degree of each node in the network. The top ranked ingredients in degree were beta-sitosterol (B1, degree = 215), stigmasterol (B2, degree = 124), rhynchophylline (C2, degree = 113), fumarine (E2, degree = 46), wogonin (HQIN2, degree = 43), 7-O-methylisomucronulatol (HQ5, degree = 40), hirsutine (C3, degree = 39), baicalein (HQIN4, degree = 35), and formononetin (HQ8, degree = 35), which may be the core components of LMQXM, while PTGS2 (degree = 86), PTGS1 (degree = 76), SCN5A (degree = 73), AR (degree = 66), ADRA1B (degree = 54), ADRB2 (degree = 52), CHRM1 (degree = 51), KCNH2 (degree = 46), CHRM3 (degree = 46), and OPRM1 (degree = 42) may be the potential targets of LMQXM.

### 3.3 Intersection targets of LMQXM and ADHD

**FIGURE 2:**
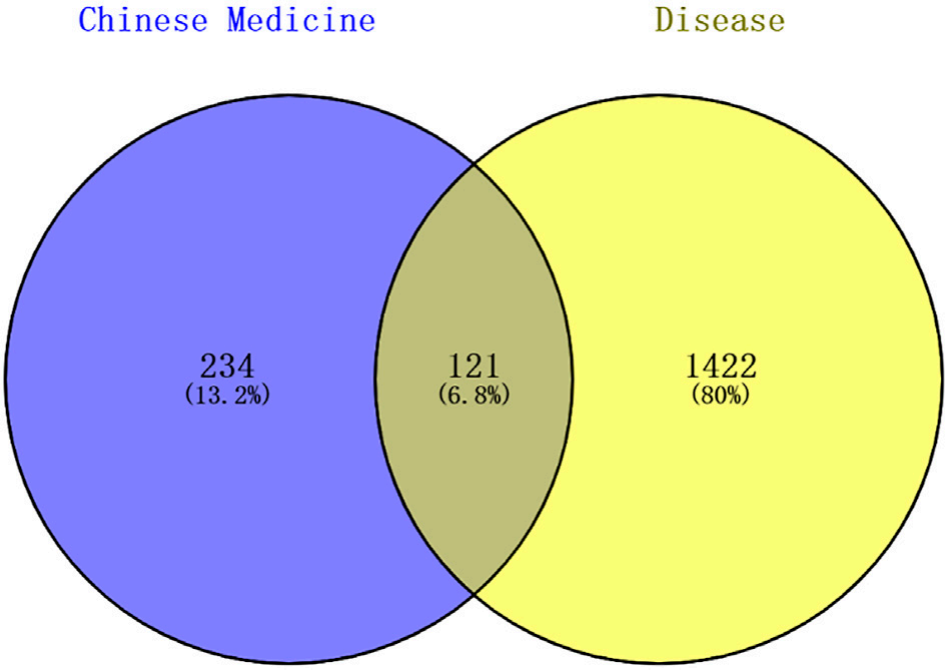
Venn diagram of Chinese medicine and disease targets. The shaded areas are the intersection targets of Chinese medicine and disease.

A total of 1543 targets relevant to ADHD were obtained from the disease databases, which included 179 targets from the DrugBank database, 873 targets from the GeneCards database (the median score >14.73 was set as the screening condition), 41 targets from TTD, and 967 targets from the DisGeNET database (Score >0.8). A Venn diagram was used to generate 121 intersection targets related to LMQXM and ADHD (Figure 2). These included SCN5A, CHRM3, CHRM1, SLC6A2, HTR3A, ADRB2, DRD1, DRD2, KCNH2, HTR2A, etc. These targets had higher degree values in the Chinese medicine-active ingredient-target network, and hence may be potential active targets of LMQXM for ADHD.

### 3.4 PPI network and core targets screening of LMQXM for ADHD

**FIGURE 3:**
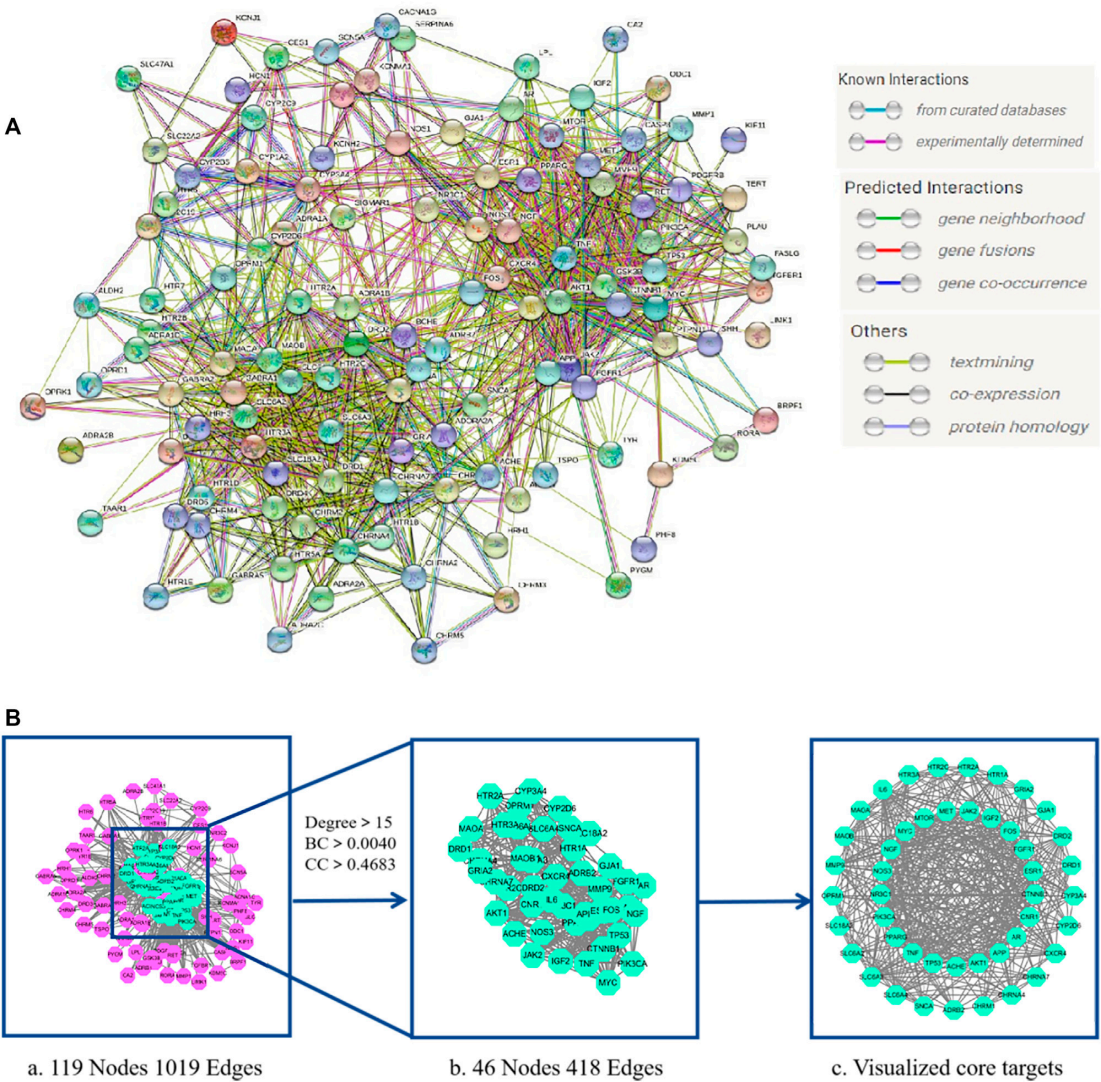
LMQXM core target screening **(A)** LMQXM and ADHD intersection target PPI network (STRING database). Network nodes represent proteins, and edges represent protein-protein associations **(B)** The core target of LMQXM for ADHD. (a) PPI network analysis of common targets between LMQXM and ADHD determined using STRING and Cytoscape. (b) 46 core targets screened based on the median of degree, BC, and CC. (c) Visualization of distribution of core targets using Cytoscape.

We uploaded the intersection targets to the STRING database and excluded the free node HRH2 and EBP. The PPI network was then generated with 119 nodes and 1019 edges with an average node degree value of 17 (Figure 3A). The PPI network “TSV” file was downloaded and imported into Cytoscape 3.7.2 to generate the core target visualization PPI network (Figure 3B). “Analyze Network” topology analysis indicated that the median degree was 15, the median of BC (Betweenness Centrality) was 0.0040, and the median of CC (Closeness Centrality) was 0.4683.46 target proteins with degree >15, BC > 0.0040, and CC > 0.4683 were screened out to generate the core targets for possible involvement of LMQXM in the treatment of ADHD (Table 5).

**TABLE 5:** The core targets of LMQXM for ADHD.

Number	Target	Description	BC	CC	Degree
1	SLC6A4	Sodium-dependent serotonin transporter	0.0750	0.6020	49
2	AKT1	RAC-alpha serine/threonine-protein kinase	0.0701	0.6146	47
3	SLC6A3	Sodium-dependent dopamine transporter	0.0633	0.5990	46
4	IL-6	Interleukin-6	0.0463	0.6020	44
5	CTNNB1	Catenin beta-1	0.0457	0.5784	41
6	FOS	Protein c-Fos	0.0506	0.5900	41
7	TP53	Cellular tumor antigen p53	0.0402	0.5413	40
8	TNF	Tumor necrosis factor	0.0165	0.5514	39
9	ESR1	Estrogen receptor	0.0270	0.5700	37
10	MYC	Myc proto-oncogene protein	0.0244	0.5488	36
11	SLC6A2	Sodium-dependent noradrenaline transporter	0.0457	0.5244	36
12	NGF	Beta-nerve growth factor	0.0149	0.5540	33
13	MAOA	Amine oxidase [flavin-containing] A	0.0141	0.5198	33
14	CHRNA4	Neuronal acetylcholine receptor subunit alpha-4	0.0206	0.5198	33
15	MAOB	Amine oxidase [flavin-containing] B	0.0151	0.5315	32
16	MTOR	Serine/threonine-protein kinase mTOR	0.0107	0.5086	30
17	CXCR4	C-X-C chemokine receptor type 4	0.0216	0.5413	30
18	DRD2	D 2) dopamine receptor	0.0254	0.5592	30
19	MMP9	Matrix metalloproteinase-9	0.0116	0.4979	29
20	HTR3A	5-hydroxytryptamine receptor 3A	0.0189	0.5364	29
21	NR3C1	Glucocorticoid receptor	0.0276	0.5592	28
22	CNR1	Cannabinoid receptor 1	0.0298	0.5438	28
23	MET	Hepatocyte growth factor receptor	0.0057	0.5021	27
24	CHRNA7	Neuronal acetylcholine receptor subunit alpha-7	0.0214	0.5244	27
25	HTR1A	5-hydroxytryptamine receptor 1A	0.0301	0.5514	27
26	HTR2A	5-hydroxytryptamine receptor 2A	0.0120	0.5438	26
27	PPARG	Peroxisome proliferator-activated receptor gamma	0.0046	0.5108	25
28	NOS3	Nitric oxide synthase	0.0147	0.5153	25
29	APP	Amyloid-beta precursor protein	0.0101	0.5315	25
30	CYP3A4	Cytochrome P450 3A4	0.0292	0.5268	24
31	SLC18A2	Synaptic vesicular amine transporter	0.0070	0.4777	24
32	ACHE	Acetylcholinesterase	0.0162	0.5291	24
33	PIK3CA	Phosphatidylinositol 4,5-bisphosphate 3-kinase catalytic subunit alpha isoform	0.0041	0.4701	22
34	HTR2C	5-hydroxytryptamine receptor 2C	0.0119	0.5130	22
35	DRD1	D (1A) dopamine receptor	0.0063	0.5021	21
36	FGFR1	Fibroblast growth factor receptor 1	0.0095	0.5021	21
37	IGF2	Insulin-like growth factor II	0.0060	0.4758	21
38	AR	Androgen receptor	0.0080	0.4876	21
39	CYP2D6	Cytochrome P450 2D6	0.0165	0.5108	21
40	JAK2	Tyrosine-protein kinase JAK2	0.0082	0.5021	21
41	CHRM1	Muscarinic acetylcholine receptor M1	0.0154	0.4917	21
42	GRIA2	Glutamate receptor 2	0.0136	0.5175	20
43	ADRB2	Beta-2 adrenergic receptor	0.0109	0.5198	18
44	SNCA	Alpha-synuclein	0.0072	0.5064	18
45	OPRM1	Mu-type opioid receptor	0.0076	0.5021	16
46	GJA1	Gap junction alpha-1 protein	0.0138	0.4816	16

### 3.5 Potential mechanism of LMQXM for ADHD

#### 3.5.1 GO enrichment analysis

**FIGURE 4:**
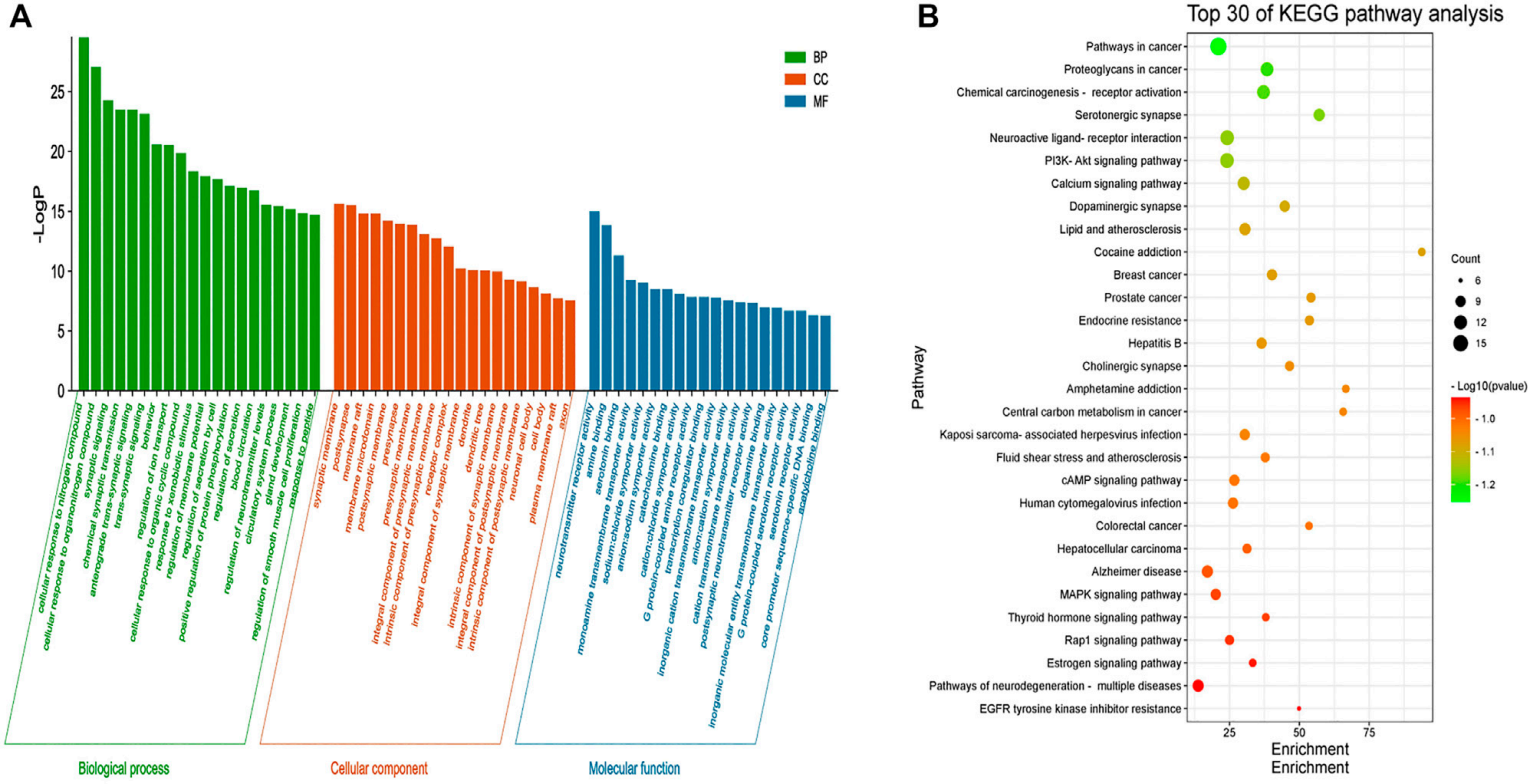
**(A)** GO enrichment analysis of the core targets of LMQXM for ADHD. The green bar chart denotes the bio-logical process (BP) enrichment analysis, the red bar chart denotes the cell composition (CC) enrichment analysis, and the blue bar chart denotes the molecular function (MP) enrichment analysis. The horizontal axis represents the name of the enrichment analysis, the vertical axis represents the negative value of LogP. The smaller the -LogP value, the more significant the enrichment of the differential genes **(B)** The top 30 pathways of KEGG enrichment analysis of LMQXM for ADHD. The abscissa represents the size of the enrichment, the ordinate represents the enriched pathway, the size of the bubble represents the number of genes in the pathway, and the color of the bubble from green to red represents the *p*-value from small to large.

GO enrichment analysis was performed on 46 core targets using the Metascape platform. A total of 1102 items were obtained (*p* < 0.01) and included 922 items for biological process, 73 items for cell composition, and 107 items for molecular function. The top 20 most significant items were selected for visual analysis based on the LogP value (Figure 4A). The results showed that the involved biological process was mainly cellular responses to nitrogen compounds, cellular response to organonitrogen compounds, synaptic signaling, chemical synaptic transmission, and anterograde trans-synaptic signaling. Cell composition was primarily related to the synaptic membrane, postsynapse, membrane raft, membrane microdomain, and postsynaptic membrane. Molecular function was mainly involved in neurotransmitter receptor activity, amine binding, serotonin binding, monoamine transmembrane transporter activity, and sodium: chloride symporter activity. These findings suggested that the mechanism of action of LMQXM for the treatment of ADHD was the result of multiple synergistic effects of multiple molecular biological processes.

#### 3.5.2 KEGG pathway enrichment analysis

To further explore the signaling pathways associated with LMQXM for ADHD, we used Metascape to identify signaling pathways enriched among the 46 core targets. A total of 134 signaling pathways were screened by KEGG pathway enrichment analysis, and some of the potential pathways related to ADHD are summarized in the (Supplementary Table S3). Based on LogP, we used the most significant top 30 pathways enriched by KEGG to generate a bubble map (Figure 4B). Based on the data and literature, enrichment of serotonergic synapse, neuroactive ligand-receptor interaction, calcium signaling pathway, dopaminergic synapse, cAMP signaling pathway, human cytomegalovirus infection, Alzheimer’s disease, MAPK signaling pathway, and pathways of neurodegeneration may be how LMQXM acts on ADHD, among which dopaminergic synapses and cAMP signaling pathway are the perspective directions for the current study of neurotransmitter transmission pathways regulating ADHD.

### 3.6 Molecular docking simulation

**FIGURE 5:**
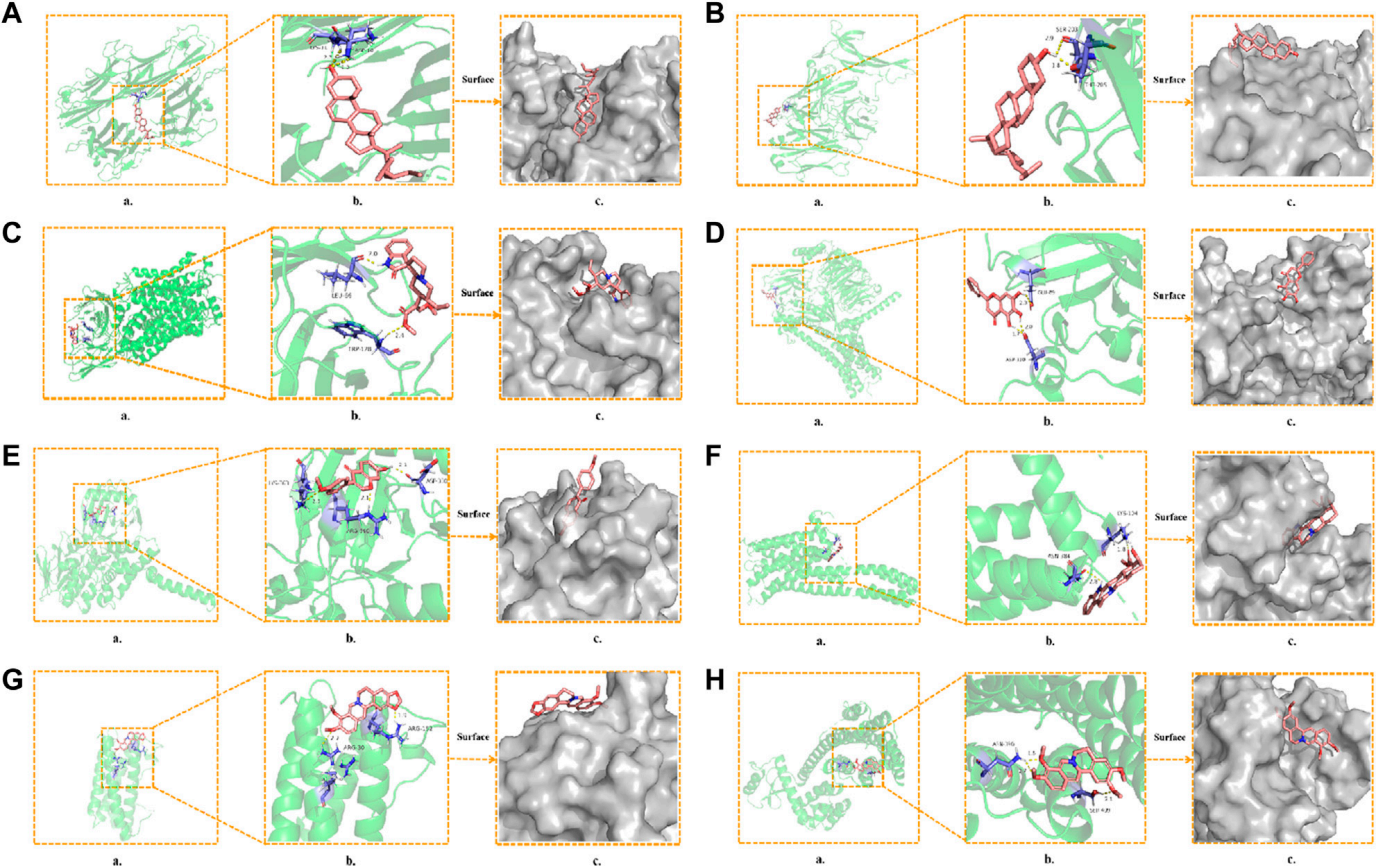
Molecular docking model diagram **(A)** Beta-sitosterol—TNF **(B)** Stigmasterol—NGF **(C)** Rhynchophyllin—SLC6A4 **(D)** Baicalein—DRD1 **(E)** Formononetin—MAOA **(F)** Yohim-bine—HTR2A **(G)** Berberine—IL6 **(H)** Palmatine—DRD2. (a) Molecular docking model, the red represents the 3D structure of the component ligand, the green represents the 3D structure of the target receptor, and the blue represents the amino acid residues (b) In the enlarged structure of the docking site, the yellow represents the hydrogen bond formed between the ligand and the amino acid residue, and the number represents the bond length (c) Combination diagram of the surface structure of the target receptor and the component ligand, the red represents the 3D structure of the ligand, and the gray represents the receptor surface structure.

To further determine the regulatory effect of LMQXM active ingredients on target proteins, we screened beta-sitosterol, stigmasterol, rhynchophylline, baicalein, formononetin, yohimbine, berberine, and palmatine active ingredients as ligands, and SLC6A4, IL-6, TNF, NGF, MAOA, MAOB, DRD1, DRD2 and HTR2A targets as receptors for molecular docking simulation based on the current status of ADHD research. Molecular docking results showed that the above 8 active ingredients could spontaneously bind to the nine targets in the natural state. The lower the binding energy, the more stable the conformation of the active ingredient binding to the target (Wei et al., 2020). Except for beta-sitosterol-MAOB and baicalein-NGF, the absolute value of the binding energy of all ligands and receptors were lower than 5 KJ/mol. The other active ingredients had high binding energy to targets, indicating that their docking results were good (Table 6). For example, beta-sitosterol was connected to the amino acid residues LYS-11 and ASP-10 of TNF through two hydrogen bonds (both with a bond length of 2.3 Å) to form a relatively stable conformation (binding energy = −20.13 kJ/mol, Figure 5A); rhynchophyllin stably bound to TRP-128 and LEU-66 amino acid residues of SLC6A4 by forming two hydrogen bonds (bond lengths are 2.4 Å and 2.0 Å, respectively) (binding energy = −18.95 KJ/mol, Figure 5C); formononetin formed three hydrogen bonds with the amino acid residues ASP-330, ARG-360, and LYS-363 of MAOA (the bond lengths were 2.1 Å, 2.1 Å, and 2.5 Å, respectively), these resulted in a more stable docking (binding en-ergy = −14.90, Figure 5E). Palmatine also formed three hydrogen bonds with amino acid residues ASN-396 and SER-409 of DRD2 (bond lengths are 1.8 Å, 2.7 Å, and 2.1 Å, respectively) for stable binding (binding energy = −14.81, Figure 5H). Our findings further proved that the above 8 compounds could be the most critical core active ingredients of LMQXM for ADHD, with mechanisms of action involving multiple processes such as neuroinflammation and neurotransmitter dysregulation (Saedisomeolia et al., 2018). For the neurotransmitter dysregulation hypothesis, the cAMP signaling pathway, as a classical signaling pathway downstream of DA, plays an important role in regulating DA content in the brain through the activation of key targets DRD1 and DRD2 (Zhou et al., 2017). Part of the molecular docking model dia-gram is shown in Figure 5.

**TABLE 6:** The binding energy of key active ingredients docked with target molecules.

Active ingredient	Target protein	PDB ID	Amino acid residue	Binding energy (KJ/mol)
Beta-sitosterol	SLC6A4	6VRH	TYR212, PHE213	−14.10
	IL6	1ALU	YLS-27	−14.48
	TNF	2AZ5	LYS-11, ASP-10	−20.13
	NGF	4EDW	—	−15.06
	MAOA	2Z5Y	ARG-369, LEU-364	−3.14
	MAOB	1S3E	GLN-216	−16.23
	DRD1	7JVP	LYS-339	−14.98
	DRD2	6CM4	—	−15.23
	HTR2A	7CW8	SER-115, TRP-200	−8.54
Stigmasterol	SLC6A4	6VRH	ARG-464	−16.90
	IL6	1ALU	LYS-27	−16.86
	TNF	2AZ5	TYR-151	−20.63
	NGF	4EDW	THR-205, SER-203	−16.40
	MAOA	2Z5Y	LYS-370	−12.84
	MAOB	1S3E	GLU-243	−20.00
	DRD1	7JVP	SER-145, THR-149	−13.72
	DRD2	6CM4	—	−24.02
	HTR2A	7CW8	HIS-70	−8.83
Rhynchophyllin	SLC6A4	6VRH	TRP-128, LEU-66	−18.95
	IL6	1ALU	ARG-30	−16.86
	TNF	2AZ5	LYS-65, PHE-144	−15.65
	NGF	4EDW	GLY-18	−13.47
	MAOA	2Z5Y	HIS-365	−8.28
	MAOB	1S3E	ARG-100	−13.56
	DRD1	7JVP	LYS-29	−10.96
	DRD2	6CM4	TRP-90	−12.22
	HTR2A	7CW8	LYS-195, LYS-191	−10.25
Baicalein	SLC6A4	6VRH	ASP-247, LEU-245, LEU-248	−12.01
	IL6	1ALU	ARG-182	−14.39
	TNF	2AZ5	GLN-47, ASP-45	−16.44
	NGF	4EDW	SER-115	−4.64
	MAOA	2Z5Y	—	−6.69
	MAOB	1S3E	LYS-81	−14.48
	DRD1	7JVP	ASP-310, GLU-89	−10.13
	DRD2	6CM4	VAL-49	−10.25
	HTR2A	7CW8	LYS-104	−10.33
Formononetin	SLC6A4	6VRH	LEU-248	−15.48
	IL6	1ALU	SRG-30, LYS-27, ARG-182	−17.03
	TNF	2AZ5	GLN-27, ASP-45, ASN-30	−17.32
	NGF	4EDW	VAL-121, ASP-208	−12.22
	MAOA	2Z5Y	ASP-330, ARG-360, LYS-363	−14.90
	MAOB	1S3E	SER-200	−14.52
	DRD1	7JVP	ASP-323	−10.92
	DRD2	6CM4	—	−10.67
	HTR2A	7CW8	ASN-354, GLU-355, ASP-356	−11.42
Yohimbine	SLC6A4	6VRH	TRY-46, ARG-117	−17.28
	IL6	1ALU	ARG-182, GLN-183	−18.87
	TNF	2AZ5	SER-147, ALA-145	−21.25
	NGF	4EDW	VAL-121, LYS-209	−15.98
	MAOA	2Z5Y	GLU-399	−19.66
	MAOB	1S3E	LYS-302	−20.33
	DRD1	7JVP	CYS-307	−15.02
	DRD2	6CM4	VAL-406, TYR-37	−15.15
	HTR2A	7CW8	LYS-104, ASN-384	−13.97
Berberine	SLC6A4	6VRH	GLN-194	−13.14
	IL6	1ALU	ARG-30, ARG-182	−13.68
	TNF	2AZ5	GLN-125	−15.52
	NGF	4EDW	LYS-13	−11.34
	MAOA	2Z5Y	VAL-244	−13.43
	MAOB	1S3E	ARG-38	−15.77
	DRD1	7JVP	LUE-11	−7.87
	DRD2	6CM4	THR-42	−11.63
	HTR2A	7CW8	LYS-1032	−12.51
Palmatine	SLC6A4	6VRH	ASN-211, ASN-217	−10.63
	IL6	1ALU	LYS-27	−13.26
	TNF	2AZ5	LYS-98	−17.24
	NGF	4EDW	SER-115	−5.06
	MAOA	2Z5Y	HIS-263	−8.12
	MAOB	1S3E	MET-280	−13.01
	DRD1	7JVP	SER-31, HIS-266	−9.96
	DRD2	6CM4	ASN-396, SER-409	−14.81
	HTR2A	7CW8	LYS-320, GLU-1004	−10.84

### 3.7 Animal experiments

#### 3.7.1 Effect of LMQXM on weight of experimental rats

As shown in Table 7, LMQXM did not affect the weight gain of rats.

**TABLE 7:** Changes in weight of rats in each group.

Group	0 week (g)	1 week (g)	2 weeks (g)	4 weeks (g)
WKY	90.28 ± 3.28	135.57 ± 3.90	189.98 ± 9.79	223.68 ± 11.30
SHR	93.65 ± 4.40	142.65 ± 5.42	191.92 ± 4.47	228.15 ± 3.47
MPH	87.38 ± 7.52	131.62 ± 5.97	180.38 ± 5.96	219.70 ± 8.19
LMQXM-LD	89.90 ± 4.39	136.08 ± 10.34	175.62 ± 12.00	224.22 ± 14.05
LMQXM-MD	85.65 ± 4.78	127.05 ± 7.92	185.68 ± 11.26	221.67 ± 13.05
LMQXM-HD	88.90 ± 5.27	136.22 ± 7.22	187.87 ± 8.92	230.37 ± 12.05

#### 3.7.2 Effect of LMQXM on the rats’ behavior based on OFT

**FIGURE 6:**
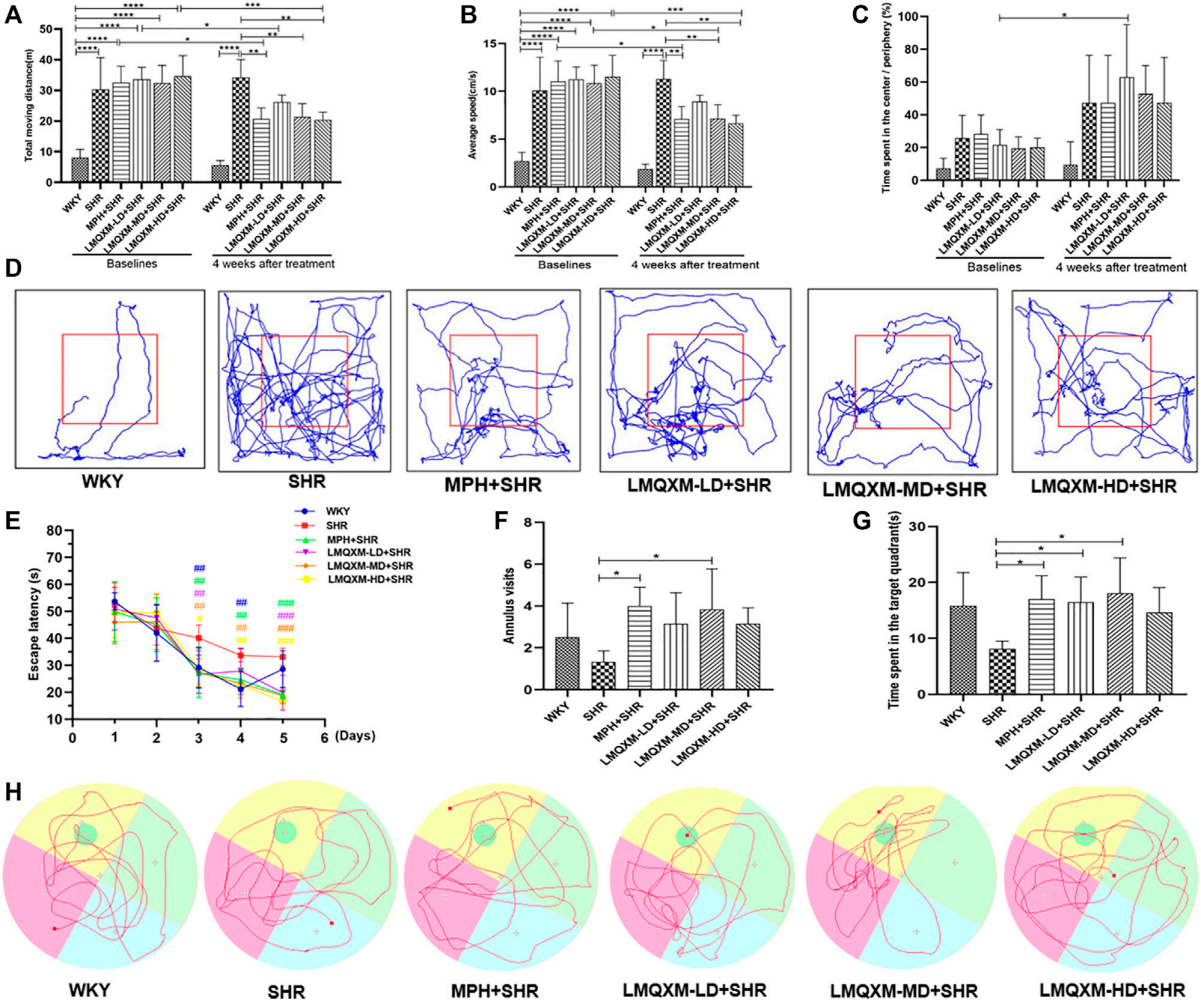
Behavioral performance of rats in each group in OFT and MWM tests **(A)** Moving distance in OFT **(B)** Average speed in OFT **(C)**. Time spent in the center/Periphery in OFT **(D)** Representative trajectories of each group of rats in OFT **(E)** Escape latency in MWM test **(F)** Number of annulus visits in MWM test **(G)** Time spent in the target quadrant in MWM test **(H)** Representative trajectories of each group in MWM test. All data are presented as mean ± SD, *n* = 6 for each group. ^*^ *p* < 0.05, ^**^ *p* < 0.01, ^***^ *p* < 0.001, ^****^ *p* < 0.0001. ^#^ *p* < 0.05, ^##^ *p* < 0.01, ^###^ *p* < 0.001 vs SHR.

The OFT was used to assess the effects of LMQXM on rats’ locomotor activity, which was evaluated by the total moving distance, average speed, and the relative proportion of time spent in the center vs periphery. The data showed that before treatment, the SHR, MPH, LMQXM-LD, LMQXM-MD, and LMQXM-HD groups had significantly more total moving distance and average speed compared with the WKY group (*p* < 0.0001, Figures 6A, B). There was no significant difference in the proportion of time spent in the center vs periphery at baseline levels in the WKY, SHR, MPH, LMQXM-LD, LMQXM-MD, and LMQXM-HD groups (Figure 6C).

After 4 weeks of drug intervention, SHRs still exhibited significantly higher total moving distance and average speed compared with WKY rats (*p* < 0.0001, Figures 6A,B), whereas total moving distance and average speed were significantly lower in the MPH, LMQXM-MD, and LMQXM-HD groups compared with the SHR group (*p* < 0.01, Figures 6A, B). Moreover, there was also a significant decrease in total moving distance and average speed in the MPH, LMQXM-MD, and LMQXM-HD groups compared with baseline levels after 4 weeks of treatment (*p* < 0.05, Figures 6A, B). After treatment, there was still no significant difference in the proportion of time spent in the center vs periphery in the WKY, SHR, MPH, LMQXM-LD, LMQXM-MD, and LMQXM-HD groups, while this ratio increased in the LMQXM-LD group after treatment compared with the baseline level (*p* < 0.05, Figure 6C). The representative movement paths of each group after administration are shown in Figure 6D.

#### 3.7.3 Effect of LMQXM on the rats’ behavior based on the MWM tests

From the third day, the escape latencies of the MPH, LMQXM-LD, LMQXM-MD, and LMQXM-HD groups were significantly shorter than those of the SHR group. WKY rats also had significantly lower escape latencies on days 3 and 4 than SHRs (*p* < 0.01, Figure 6E). In the probe trials, although the annulus visits appeared to increase in the four treatment groups compared with the SHR group, only the MPH and LMQXM-MD groups had significant differences vs the SHR group (*p* < 0.05, Figure 6F). In addition, the MPH, LMQXM-LD, and LMQXM-MD groups also spent more time in the target quadrant than the SHR group (*p* < 0.05, Figure 6G). However, there was no significant difference in the annulus visits and time spent in the target quadrant between WKY rats and SHRs in probe trials (Figures 6F, G). The representative trajectories of each group are shown in Figure 6H.

#### 3.7.4 Effect of LMQXM on DA and cAMP levels in PFC and striatum

**FIGURE 7:**
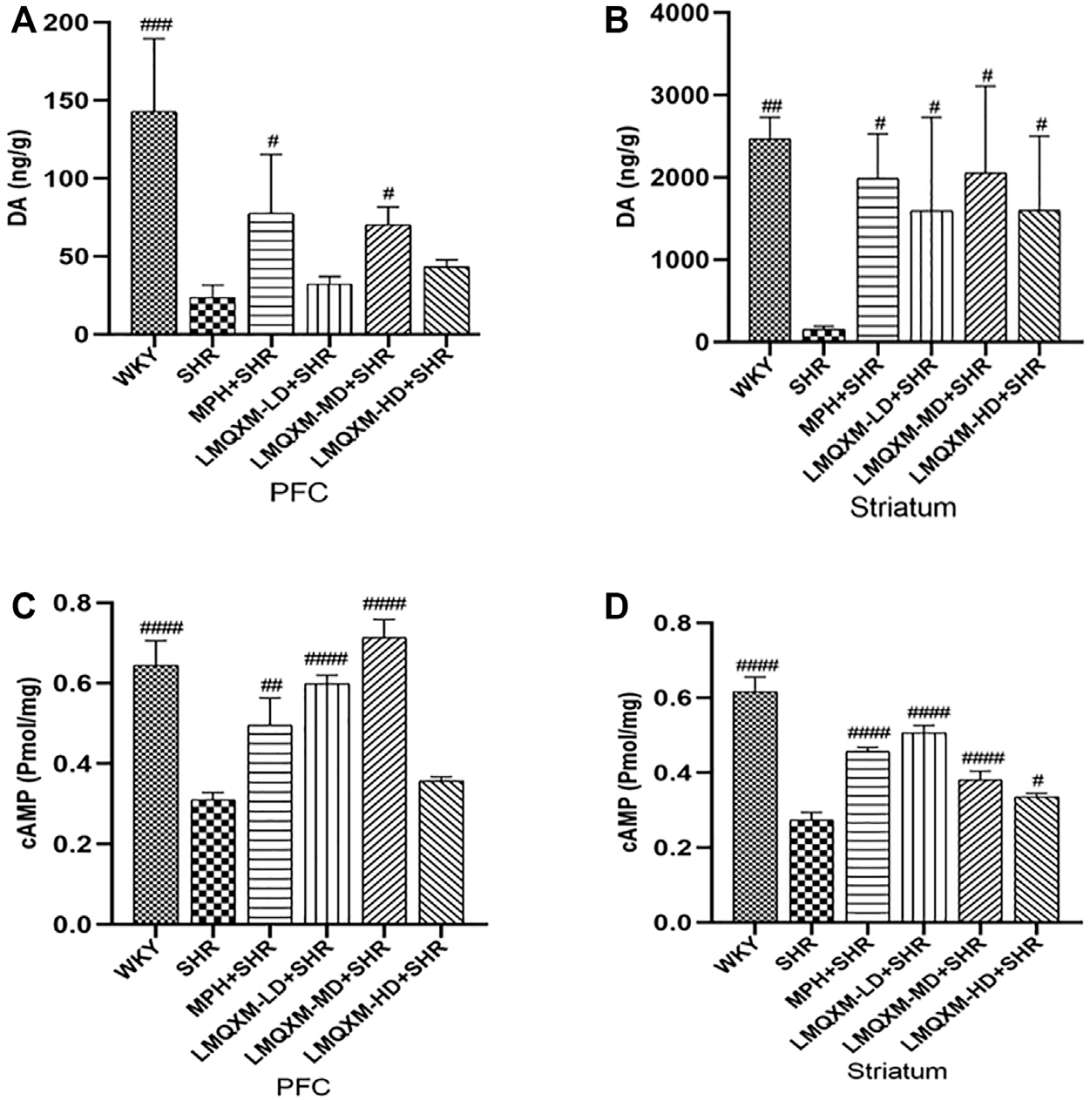
DA and cAMP levels in PFC and striatum for each group of rats **(A)**. DA levels in PFC **(B)**. DA levels in P striatum **(C)**. cAMP levels in PFC **(D)**. cAMP levels striatum. All data are presented as mean ± SD, *n* = 3 for each group. ^#^ *p* < 0.05, ^##^ *p* < 0.01, ^###^ *p* < 0.001 vs SHR.

DA is an important neurotransmitter in the brain that regulates movement, learning, and memory. cAMP is an important intracellular second messenger produced by DA GPCRs acting on adenylate cyclase (AC) to hydrolyze ATP. LC-MC and ELISA were used to detect DA and cAMP levels in brain tissue, respectively (Wang et al., 2019; Yu et al., 2022). The levels of DA and cAMP in the PFC and striatum were significantly lower in SHRs compared with WKY rats (*p* < 0.01, Figures 7A–D). After treatment, DA and cAMP levels in the PFC and striatum were significantly increased in the MPH and LMQXM-MD groups compared with the SHR group (*p* < 0.05, Figures 7A–D); meanwhile, LMQXM-HD upregulated DA and cAMP levels in the striatum of SHRs, while LMQXM-LD upregulated DA levels in the striatum and cAMP levels in the PFC of SHRs (*p* < 0.05, Figures 7B–D).

#### 3.7.5 Effect of LMQXM on MOD of DRD1, DRD2, cAMP, and PKA in PFC and striatum

**FIGURE 8:**
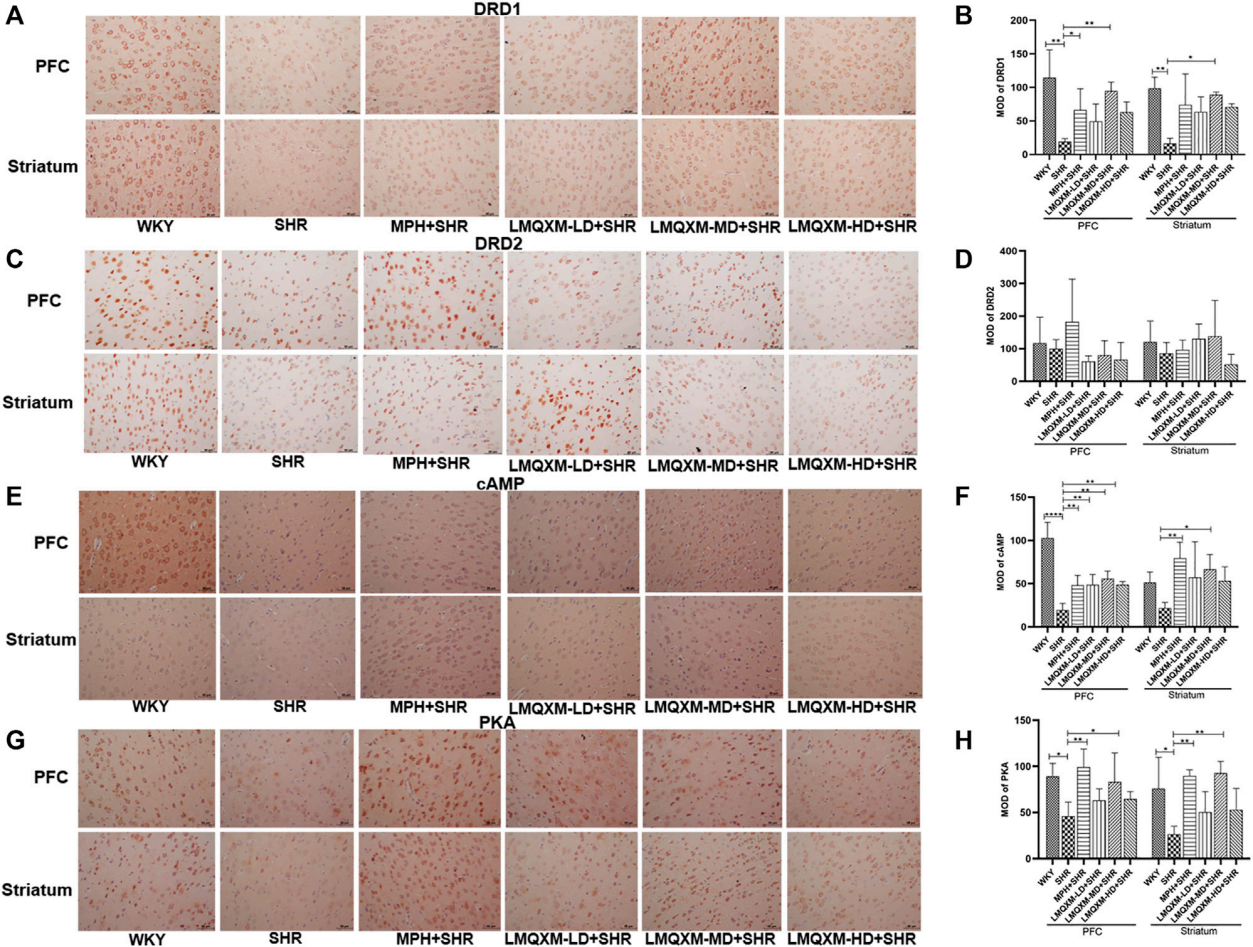
Immunohistochemical index results in PFC and striatum for each group of rats **(A)** DRD1 positive expression image from PFC and striatum **(B)** MOD quantification from DRD1 in PFC and striatum **(C)** DRD2 positive expression image from PFC and striatum **(D)** MOD quantification from DRD1 in PFC and striatum **(E)** cAMP positive expression image from PFC and striatum **(F)** MOD quantification from cAMP in PFCand striatum **(G)** PKA positive expression image from PFC and striatum **(H)** MOD quantification from PKA in PFC and striatum. The positive expression is brown yellow, and all data are presented as mean ± SD, *n* = 3 for each group. ^*^ *p* < 0.05, ^**^ *p* < 0.01, ^***^ *p* < 0.001, ^****^ *p* < 0.0001.

cAMP and PKA are DA downstream effectors that can have opposite effects on the cAMP/PKA signaling pathway when DA is coupled to DRD1 and DRD2, with an increase in cAMP most consistent with an increase in DRD1 activity or a decrease in DRD2 (Schmidt et al., 1998). They are also generally expressed in different populations of striatal medium spiny neurons (direct vs indirect pathways) and cortical cells. To assess the effect of LMQXM in regulating the cAMP/PKA signaling pathway, immunohistochemistry was used to assess the MOD of DRD1, DRD2, cAMP, and PKA in the PFC and striatum. As shown in Figure 8, we observed lower expression of brownish-yellow positive cells for DRD1, cAMP, and PKA in the PFC and striatum of SHRs than in WKY rats (Figures 8A, B, E–H). MPH and LMQXM-MD administration significantly reversed the MOD of cAMP and PKA in the PFC and striatum of SHRs (*p* < 0.05, Figures 8E–H). Furthermore, MPH also upregulated the MOD of DRD1 in the PFC of SHRs, and LMQXM-MD upregulated the MOD of DRD1 in the PFC and striatum of SHRs (*p* < 0.05, Figures 8A, B). Interestingly, we also observed that LMQXM-LD and LMQXM-HD significantly upregulated the MOD of cAMP in the PFC of SHRs (*p* < 0.01, Figures 8E, F). However, we did not find significant differences in the expression of DRD2-positive cells in the PFC and striatum of SHRs and WKY rats, nor did MPH and LMQXM administration have significant effects on the MOD expression of DRD2 in the PFC and striatum of SHRs.

#### 3.7.6 Effect of LMQXM on DRD1, DRD2, and PKA mRNA in PFC and striatum

**FIGURE 9:**
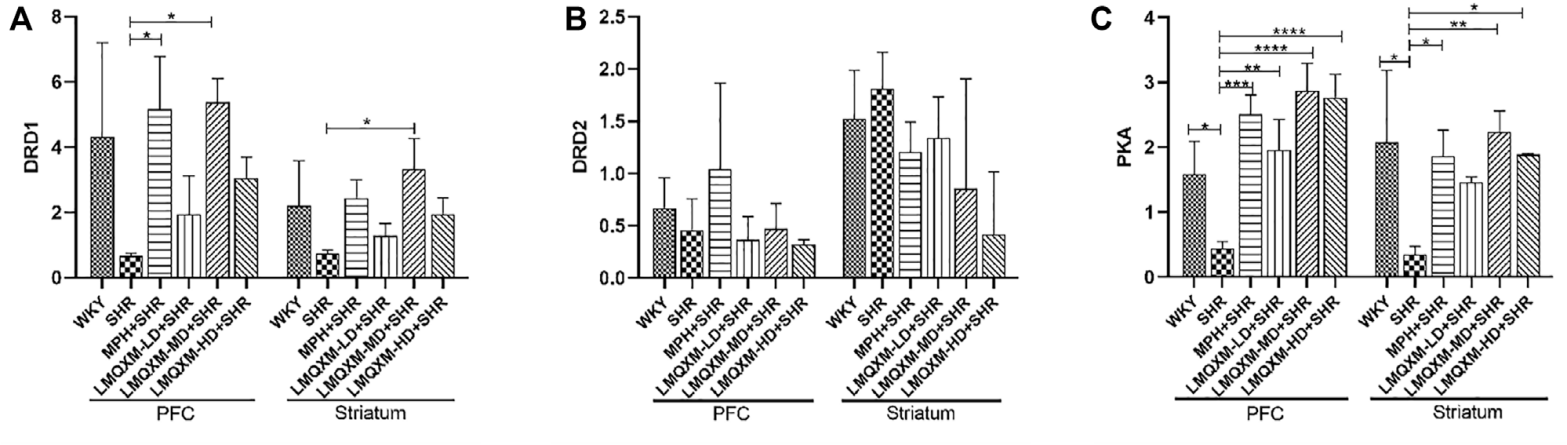
The mRNA expression of DRD1, DRD2, and PKA in PFC and striatum **(A)** DRD1 mRNA expression **(B)** DRD2 mRNA expression **(C)** PKA mRNA expression. All data are presented as mean ± SD, *n* = 3 for each group. ^*^ *p* < 0.05, ^**^ *p* < 0.01, ^***^ *p* < 0.001, ^****^ *p* < 0.0001.

To further evaluate the regulatory effect of LMQXM on the cAMP/PKA signaling pathway, qPCR was used to assess DRD1, DRD2, and PKA mRNA expression in the PFC and striatum. As shown in Figure 9, there was a trend toward reduced DRD1 mRNA expression in the PFC and striatum in the SHR group compared with the WKY group, but there was no statistical difference (Figure 9A). DRD1 mRNA expression was significantly increased in the PFC of the MPH and LMQXM-MD groups compared with the SHR group, and DRD1 mRNA expression was also significantly increased in the striatum of the LMQXM-MD group (*p* < 0.05, Figure 9A). As for the expression of DRD2 mRNA, there was no significant difference between each group for either the PFC or striatum (Figure 9B). The expression of PKA mRNA in the PFC and striatum of SHRs was significantly lower than that of WKY rats (*p* < 0.05, Figure 9C). Compared with the SHR group, PKA mRNA expression in the PFC and striatum was significantly increased in the MPH, LMQXM-MD, and LMQXM-HD groups, whereas the expression of PKA mRNA was elevated in the LMQXM-LD group only in the PFC (*p* < 0.05 Figure 9C).

## 4 Discussion

Although MPH can effectively alleviate the symptoms of ADHD, it is often associated with certain adverse effects, including growth restriction, sleep disturbances, and cardiac effects (Espadas et al., 2018; Liang et al., 2018). In addition, its treatment compliance is suboptimal (Ishizuya et al., 2021), so it is necessary to find effective and suitable therapeutic regimens for pediatric use. In this study, we used network pharmacology and molecular docking to predict the potential mechanisms of LMQXM for the treatment of ADHD and performed animal experiments to validate the role of LMQXM on the behavioral performance of ADHD model rats and the mechanisms of the cAMP/PKA signaling pathway, which provide new insights and a basis for clinical treatment and pharmacological studies of ADHD.

The degree to which top ranking components in network pharmacology, such as beta-sitosterol, stigmasterol, rhynchophylline, hirsutine, baicalein, and formononetin, have been shown to have potential advantages for the treatment of neurological disorders, consistent with the composition identified by UPLC-Q-TOF-MS. Beta-sitosterol has neuroprotective, anxiolytic, sedative, immunomodulatory, and anti-inflammatory effects (Panayotis et al., 2021), which allowed it to reduce inhibitory stress in the PFC and dentate and contextual fear memory of mice (Zahiruddin et al., 2020) and increase the levels of brain-derived neurotrophic factor (BDNF) in the hippocampus, all of which are beneficial for improving cognitive, learning and memory functions (Alzoubi et al., 2017). According to recent research, stigmasterol has anxiolytic and anticonvulsant properties *via* the positive regulation of γ-aminobutyric acid receptors, and is thus considered a new candidate steroid drug for the treatment of neurological diseases (Karim et al., 2021). Animal experiments have shown that stigmasterol could increase swimming time in the target quadrant of vanadium-induced BALB/c mice in the MWM test, resulting in improved neurocognitive and motor function (Adebiyi et al., 2018). Rhynchophylline, the main indole alkaloid isolated from GT, can pass through the blood-brain barrier relatively quickly and effectively regulate calcium and potassium channels to exert a protective effect on the nervous system (Geetha and Ramachandran, 2021). Rhynchophylline has been shown to activate the cAMP signaling pathway and MAPK/NF-κB signaling pathway to exert neurotransmitter modulation, anti-inflammatory, antioxidant, sedative, anti-Alzheimer’s disease, and anti-drug addiction effects (Geetha and Ramachandran, 2021; Qin et al., 2021). Furthermore, it can effectively rescue the spatial learning and memory deficits of rats in MWM tests and prevent and repair synaptic plasticity damage (Yang et al., 2018). Other ingredients such as berberine, hirsutine, and palmatine can also improve cognition and learning through neuroprotective effects (Sadeghnia et al., 2017; Wang et al., 2020b; Kushida et al., 2021). Baicalein has been shown to regulate motor ability and learning and memory function in ADHD model rats to improve the core symptoms of ADHD (Zhou et al., 2017).

PPI networks and topological property functions identified possible core targets for LMQXM treatment of ADHD, mainly involving the dopaminergic system (such as DRD1, DRD2, SLC6A3, MAOA, and MAOB), noradrenergic system (such as SLC6A2), and serotonergic system (such as SLC6A4, HTR1B, HTR1A, HTR2A, HTR3A, and HTR2C), whose mechanisms of action are closely related to the transmission of monoamine neurotransmitters, most of which have been classified as risk genes for ADHD (Lasky-Su et al., 2008; Banaschewski et al., 2010). The methylation status of SLC6A4 has been shown to be significantly associated with hyperactive and impulsive symptoms in ADHD (Park et al., 2015). DRD1 and DRD2 may contribute to the improvement of working memory and cognitive function in children with ADHD (Trampush et al., 2014). SLC6A3, the gene encoding DAT, is associated with the development of several psychiatric disorders, and deletion of this gene can cause mice to exhibit hyperactive and impulsive-like symptoms; therefore, DAT knockout mice are also frequently used as a classical animal model for studying ADHD (Salatino-Oliveira et al., 2018). Moreover, targets related to neuronal plasticity in ADHD (such as CHRNA4, CHRNA7, NGF, FOS, and MTOR) and targets related to neuroinflammation (such as IL-6, TNF, and MMP9) were also identified. The higher degree values of AKT1, TP53, ESR1, and MYC make them promising potential targets for LMQXM in the treatment of ADHD, but no studies have clearly shown their relevance to the pathogenesis of ADHD. AKT1 is involved in chronic neuroinflammation (Xiang et al., 2016), and TP53 has been shown to be associated with DA neuronal deformation (Kamath et al., 2022), so future studies should perhaps focus on their value in ADHD neuroinflammation and DA neuronal damage.

The most promising pathways for LMQXM treatment of ADHD, according to GO and KEGG pathway enrichment analysis, are serotonergic synapse, neuroactive ligand-receptor interaction, the PI3K-Akt signaling pathway, the calcium signaling pathway, dopaminergic synapse, the cAMP signaling pathway, Alzheimer disease, the MAPK signaling pathway, and so on. It is worth mentioning that dopaminergic synapses and the cAMP signaling pathway are the classical signaling pathways in ADHD, and they are also the current hot directions for studying neurotransmitter dysregulation in ADHD.

Dopamine deficiency or dysregulation of dopaminergic synaptic transmission mediates behavioral abnormalities or memory deficits in ADHD (Yan and Rein, 2022). cAMP is a DA downstream effector that mediates chemical or physiological responses produced by a variety of intracellular neurotransmitters (Zaccolo et al., 2021), and its expression is regulated by the opposite effects of DRD1 and DRD2. DRD1 activates adenylate cyclase (AC) by coupling to Gαs and Gαolf subtypes of the G protein α subunit, catalyzing ATP hydrolysis to induce cAMP production (Rangel Barajas et al., 2015), which further acts on PKA regulatory subunits to promote phosphorylation of a range of substrates, thereby regulating the expression of neurotransmitters and neurotrophic factors in the brain (Jichao et al., 2017). DRD2 inhibits AC activity and negatively regulates cAMP production by coupling to Gαi/o of the G protein α subunit (Thomas Broome et al., 2020). The cAMP/PKA signaling pathway may feedback affect the stabilization of DA content in brain tissue through the synergistic effect of DRD1 and DRD2. In the molecular docking, we found that the core components of LMQXM all bound well to DRD1 and DRD2. To investigate the effect of the cAMP/PKA signaling pathway on DA levels and behavioral disorders in ADHD model rats, we observed the possible mechanism of LMQXM action through animal experiments.

In this study, we used SHRs as the animal model of ADHD. SHR presents symptoms similar to ADHD between 4 and 10 weeks of age, and is currently the most widely studied animal model of ADHD, whereas WKY rats are often used as natural controls (Rahi and Kumar, 2021). We found that LMQXM does not affect rats’ weight gain, suggesting the drug’s safety. OFT and MWM tests were used to assess the effects of LMQXM on the behavioral performance of experimental rats, including activity and learning and memory (Yuan et al., 2019). Consistent with previous studies, SHRs exhibited significantly increased total moving distance and average speed, which represent typical symptoms of hyperactivity. After treatment, the total moving distance and average speed were significantly lower in the MPH, LMQXM-MD, and LMQXM-HD groups vs the SHR group, indicating that MPH, medium and high doses of LMQXM effectively improved hyperactive behavior in SHRs. There was no significant difference between the WKY and SHR groups in the ratio of time spent in the center to time spent in the periphery, and no significant change after treatment, suggesting no difference in anxiety levels between the two. It has been suggested that WKY rats often show depressive and anxious behaviors in behavioral tests (Nam et al., 2014), but our results showed that WKY rats did not show significant anxiety compared to SHR rats. In the future, SD rats should be added as controls to compare the anxiety status of the three. The WKY, MPH, LMQXM-LD, LMQXM-MD, and LMQXM-HD groups showed significantly shorter escape latency from day 3 compared with the SHR group, indicating the learning and memory deficit in SHRs that could be reversed by MPH and LMQXM. In addition, the annulus visits and time spent in the target quadrant were significantly higher in the MPH and LMQXM-MD groups compared to the SHR group, suggesting that MPH and LMQXM-MD are more advantageous for improving learning and memory in SHRs. In conclusion, MPH and LMQXM-MD were most effective in reversing behavioral disorders in SHRs, while LMQXM-HD only improved hyperactivity performance, which provides a basis for LMQXM to improve the core symptoms of ADHD as well as dose reference.

The PFC receives projections from midbrain DA neurons, which in turn project to other DAergic regions such as the striatum, resulting in a complex neuromodulatory system that is critical for many motor, cognitive, and motivational processes (Chau et al., 2018; Adrover et al., 2020), which is a key region for studying behavioral cognitive disorders. The synthesis and release of DA in the PFC or striatum are regulated bidirectionally by the cAMP/PKA signaling pathway (Nishi et al., 2008), a key pathway that mediates DA transmission and thus performs motor, learning, and memory functions (Nishi and Snyder, 2010). Studies have shown that the cAMP/PKA signaling pathway is closely related to the formation of DA defects in ADHD. The short chain portion of DRD2 activated by DA in the synaptic gap can inhibit the cAMP/PKA signaling pathway, which in turn feedbacks to suppress DA synthesis and reduce the rate of DA synthesis, ultimately leading to the DA-deficient state in the brain (Zhou et al., 2017). DAT-CI mice have hyperactive symptoms, and amphetamine enhances striatal DAergic neurotransmission and inhibits paradoxical movements in DAT-CI mice by a mechanism related to DRD1/cAMP/PKA/DARPP32 pathway activation (Napolitano et al., 2010). In addition, a significant DRD1/DRD2 imbalance was found in the brain tissue of SHRs, and the cAMP/PKA signaling pathway was in a downregulated state; tomoxetine (a non-neuroleptic stimulant for ADHD) or Jingning granules (an herbal prescription) rescued the DRD1/DRD2 imbalance by activating the cAMP/PKA signaling pathway, thereby improving behavioral and cognitive function in SHRs (Ding et al., 2022). In the present study, we measured the expression of DA, cAMP, DRD1, DRD2, and PKA in rat PFC and striatum. The DA levels in the PFC and striatum were significantly lower in SHRs compared with WKY rats, indicating that DA deficiency is the main mechanism of behavioral disorders in SHRs, which is consistent with previous reports in the literature. After treatment, the levels of DA in the PFC and striatum were significantly higher in the MPH and LMQXM-MD groups compared with the SHR group, indicating that MPH and LMQXM-MD rescued the behavioral disorder of SHRs by elevating the levels of DA in the PFC and striatum. In addition, LMQXM-LD and LMQXM-HD upregulated DA levels in the striatum of SHRs but had no significant effect on DA levels in the PFC, considering that this may be closely related to the relatively sparse distribution of DA neurons in the PFC, the low number of uptake sites, and the high DA turnover rate (Goto et al., 2007).

As previously described, the cAMP/PKA signaling pathway is subject to opposite regulation by DRD1 and DRD2, which in turn affects DA production in a feedback manner. In the present study, we found that SHRs had significantly lower cAMP levels and cAMP-positive cell expression in the PFC and striatum than WKY rats, suggesting that the SHRs’ brain tissue cAMP pathway may be in a downregulated state, resulting in the DA-deficient state. Simultaneously, the expression of PKA and DRD1 brown yellow-stained particles, as well as PKA mRNA, was significantly downregulated in the PFC and striatum of SHRs, indicating that inactivation of DRD1 and the cAMP/PKA signaling pathway in SHRs resulted in behavioral manifestations similar to those of ADHD. MPH and LMQXM-MD increased the cAMP level and positive cell expression of SHRs in the PFC and striatum and upregulated the expression of PKA and DRD1 positive cells and mRNA in the PFC and striatum of SHRs, but had no significant effect on DRD2. In addition, LMXQM-LD and LMQXM-HD also upregulated striatal cAMP levels, cAMP-positive cell expression in the PFC, and PKA mRNA expression in the PFC of SHRs, indicating that LMQXM activates the cAMP/PKA signaling pathway mainly through activation of DRD1, which in turn elevates DA levels in the PFC and striatum, thereby alleviating SHRs’ hyperactivity and learning and memory deficits, which is consistent with a model in which SHRs exhibit a DRD1/AC/cAMP/PKA signaling defect that can be rescued with MPH or LMQXM without significant effects on D2-expressing cells.

Our study initially predicted the potential mechanism of LMQXM for the treatment of ADHD and verified through animal experiments that LMQXM mainly acts on DRD1 to regulate the cAMP/PKA signaling pathway to increase DA levels in the PFC and striatum to control the behavioral disorders of SHRs and is most effective at medium doses. Low and high doses were not as effective as medium doses, and we believed that this mixture has complex components, more targets, different affinity effects, and is affected by factors such as intestinal epithelial absorption rate and blood-brain barrier passage rate, thus producing different effects from medium doses.

Our study has some limitations: firstly, the sample size of the animal experiment was low due to the limitation of experimental funding, which may have some bias on the expression of the results; secondly, although WKY rats are often used as natural controls for SHRs, the addition of SD rats as a control would make the results more convincing; finally, the study did not conduct the DAT, the target of first-line drugs for the treatment of ADHD, to in-depth study was not conducted to compare with MPH to further reveal the mechanism of DA neurotransmission. In future studies, we will further expand the sample size, add SD rats as a control, further investigate the effect of LMQXM on the behavioral performance of SHRs and the effect of DAT, and focus on the possible role of DRD2. In addition, the role of ADHD neuroinflammation will also be a key direction for future research.

In conclusion, our study provided a preliminary explanation the centrality of DA deficiency in the pathogenesis of ADHD and that the specific binding of DA and DRD1 elevates DA levels in the PFC and striatum by activating the cAMP/PKA signaling pathway, which may be an effective way to improve the core symptoms of ADHD.

## Data Availability

The datasets presented in this study can be found in online repositories. The names of the repository/repositories and accession number(s) can be found in the article Supplementary Material.
