# Supplementary material for: Network pharmacology, molecular docking, and experimental validation to explore the potential mechanism of Long Mu Qing Xin mixture for the treatment of attention deficit hyperactivity disorder
Source: Front Pharmacol. 2023 Mar 17;14:1144907. doi: 10.3389/fphar.2023.1144907 (PMC10063801; doi:10.3389/fphar.2023.1144907)
Supplement: Supplementary file 1 [file Presentation1.pdf]

Supplementary Material

Network Pharmacology, Molecular Docking and Experimental Validation to Explore the Potential Mechanism of Long Mu Qing Xin Mixture for the treatment of Attention Deficit Hyperactivity Disorder

Xuejun Li, Zhen Xiao*, Wenyan Pu, Zhiyan Jiang*, Shumin Wang, Yixing Zhang

*** Correspondence:** Zhen Xiao: xiaozhen@shutcm.edu.cn; Zhiyan Jiang: lhjzycm@163.com

1 Supplementary Tables

Supplementary Table 1 Identification of chemical composition of LMQXM

Number	Time （min）	Additive ion	*m/z*	ppm	Formula	Component Name	MS/MS
1	3.44	$[M-H]^-$	169.0143	0.3	$C_7H_6O_5$	Gallic acid	169.0139;125.0244;97.0291;79.0191
2	4.74	$[M+H]^+$	268.1031	-3.5	$C_{10}H_{13}N_5O_4$	Adenosine	268.1013;136.0616;119.0353
3	5.96	$[M+H]^+$	330.0603	1.5	$C_{10}H_{12}N_5O_6P$	Cyclicadenosine monophosphate	330.0606;136.0615;119.0364
4	11.64	$[M-H]^-$	543.1179	9.7	$C_{26}H_{24}O_{13}$	Hyemaloside B	543.1174;421.0799;259.0276;121.0297
5	12.97	$[M-H]^-$	367.1049	3.9	$C_{17}H_{20}O_9$	3-O-Feruloylquinic acid	367.1030;285.0773;193.0506;134.0376
6	14.73	M^+	342.1706	1.8	$C_{20}H_{24}NO_4^+$	Phellodendrine	342.1687;192.1014;177.0780;148.0751
7	15.49	$[M+FA-H]^-$	525.1602	-2.2	$C_{23}H_{28}O_{11}$	Albiflorin	525.1596;479.1533;357.1179;283.0801;121.0295
8	16.10	M^+	342.1701	-0.5	$C_{20}H_{24}NO_4^+$	Magnoflorine	342.1705;297.1126;265.0863;237.0905
9	16.41	$[M+FA-H]^-$	525.1625	2.2	$C_{23}H_{28}O_{11}$	Paeoniflorin	525.1613;479.1555;449.1444;327.1074;165.05556;121.0291
10	17.42	$[M-H]^-$	193.0504	-1.2	$C_{10}H_{10}O_4$	Ferulic acid	178.0278;134.0377
11	18.45	$[M-H]^-$	417.1195	0.9	$C_{21}H_{22}O_9$	Liquiritin	417.1188;255.0659;135.0091;119.0503
12	18.73	$[M-H]^-$	547.1456	-0.2	$C_{26}H_{28}O_{13}$	Chrysin 6-C-β-L-arabinoside-8-C-β-D-glucoside	547.1430;529.1329;487.1222;457.1118;367.0803;337.0697

13	18.81	[M-H]-	405.118	-2.7	$C_{20}H_{22}O_9$	2,3,5,4'-Tetrahydroxystilbene 2-O-glucoside	405.1179;243.0665;225.0570;173.0614
14	19.14	[M-H]-	631.1669	0.1	$C_{30}H_{32}O_{15}$	Galloylpaeoniflorin	631.1667;465.1396;313.0551;169.0137
15	19.39	[M-H]-	547.1457	0.0	$C_{26}H_{28}O_{13}$	Chrysin 6-C-β-D-glucoside-8-C-β-L-arabinoside	547.1433;457.1129;427.1018;367.0814;337.0709
16	20.79	[M+FA-H]-	525.1604	-1.8	$C_{23}H_{28}O_{11}$	Mudanpioside I	525.1610;479.1548;357.1177;327.1063;283.0818;121.0292
17	21.09	[M+H]+	385.2113	-2.3	$C_{22}H_{28}N_2O_4$	Isorhynchophylline	385.2132;353.1871;241.1341;160.0762
18	21.32	[M+FA-H]-	507.1491	-0.6	$C_{23}H_{26}O_{10}$	Lactiflorin	507.1502;461.1450;339.1073;177.0558;121.0302
19	22.13	[M-H]-	445.0764	-2.8	$C_{21}H_{18}O_{11}$	Baicalin	445.0777;269.0456;241.0510;223.0399.195.0447
20	22.22	[M+H]+	385.213	2.1	$C_{22}H_{28}N_2O_4$	Rhynchophylline	385.2117;353.1853;269.1639;215.1174;160.0758
21	22.73	[M-H]-	255.0654	-3.5	$C_{15}H_{12}O_4$	Liquiritigenin	255.0665;135.0097;119.0505
22	22.97	[M-H]-	165.0553	-2.5	$C_9H_{10}O_3$	Paeonol	165.0560;137.0247;93.0350;92.0273
23	23.26	[M-H]-	445.0766	-0.3	$C_{21}H_{18}O_{11}$	Norwogonin 7-O-glucuronide	445.0786;269.0433;225.0542;197.0597
24	23.48	M+	336.1236	1.7	$C_{20}H_{18}NO_4^+$	Berberine	336.1232;320.0921;304.0976;292.0973;278.0819
25	23.76	[M-H]-	283.0616	1.4	$C_{16}H_{12}O_5$	Calycosin	283.0594;268.0354;211.0383;184.0508;135.0075
26	24.03	[M-H]-	459.0923	-2.1	$C_{22}H_{20}O_{11}$	Oroxylin A 7-O-glucuronide	459.0921;283.0614;268.0370;175.0253;113.0249
27	24.40	[M-H]-	431.0979	-1.1	$C_{21}H_{20}O_{10}$	Emodin-8-glucoside	431.0954;269.0426;225.0543
28	24.63	[M-H]-	459.0938	1.1	$C_{22}H_{20}O_{11}$	Wogonin 7-O-glucuronide	459.0926;283.0609;268.0368;175.0256;113.0255
29	24.94	[M+H]+	355.2037	5.9	$C_{21}H_{26}N_2O_3$	Yohimbine	355.2047;337.1968;224.1296;212.1282;144.0818
30	25.75	[M+FA-H]-	629.1883	1.1	$C_{30}H_{32}O_{12}$	Benzoylpaeoniflorin	629.1908;583.1853;553.1739;431.1354;121.0303
31	26.89	[M-H]-	269.0457	0.6	$C_{15}H_{10}O_5$	Baicalein	269.0440;251.0338;241.0496;223.0385;195.0650;136.9882
32	28.26	[M-H]-	267.0673	3.8	$C_{16}H_{12}O_4$	Formononetin	267.0666;252.0428;223.0406;195.0452;132.0224
33	28.96	[M-H]-	819.3801	-0.9	$C_{42}H_{60}O_{16}$	Licoricesaponin E2	819.3811;351.0555;193.0350
34	29.12	[M-H]-	837.3927	1.3	$C_{42}H_{62}O_{17}$	Licoricesaponin G2	837.3916;351.0560;193.0352;175.0244

35	29.53	$[M+FA-H]^-$	829.4589	-0.3	$C_{41}H_{68}O_{14}$	Astragaloside IV	829.4543;783.4487;621.4047;489.3607;445.0764
36	30.06	$[M+FA-H]^-$	515.1915	-1.5	$C_{26}H_{30}O_8$	Obaculactone	515.1954;469.1886;411.1451;381.2093;306.1264;229.1238
37	30.53	$[M-H]^-$	821.3951	-1.7	$C_{42}H_{62}O_{16}$	Glycyrrhizic acid	821.3947;351.0557;193.0350;175.0244
38	31.89	$[M+H]^+$	433.2237	3.7	$C_{24}H_{32}O_7$	Schisandrol A	415.2124;400.1894;384.1938;369.1700
39	35.64	$[M-H]^-$	397.2009	-2.9	$C_{24}H_{30}O_5$	(3Z,5aS,6S,9S,9aS)-3-Butylidene-1,3,4,5,5a,6,9,9a-octahydro-1-oxo-9-(1-oxopentyl)-6,9-ethanonaphtho[1,2-c]furan-8-carboxylic acid	397.2015;353.2116;207.1032;189.0923;163.1131
40	38.29	$[M+H]^+$	417.2272	0.1	$C_{24}H_{32}O_6$	Schisandrin A	417.2298;402.2064;347.1493;316.1316;301.1076

Supplementary Table 2 Detailed information on the 164 active compounds derived from LMQXM.

Mol ID/CAS	Number	Mol Name	OB/GIA	BBB	DL	Related Chinese medicine
MOL000211	A1	Mairin	55.38	0.22	0.78	HQ, DZ, BS
MOL000239	HQ1	Jaranol	50.83	-0.22	0.29	HQ
MOL000296	HQ2	Hederagenin	36.91	0.96	0.75	HQ
MOL000033	HQ3	(3S,8S,9S,10R,13R,14S,17R)-10,13-dimethyl-17-[(2R,5S)-5-propan-2-yloctan-2-yl]-2,3,4,7,8,9,11,12,14,15,16,17-dodecahydro-1H-cyclopenta[a]phenanthren-3-ol	36.23	1.09	0.78	HQ
MOL000371	HQ4	3,9-di-O-methylnissolin	53.74	0.63	0.48	HQ
MOL000378	HQ5	7-O-methylisomucronulatol	74.69	0.84	0.3	HQ
MOL000380	HQ6	(6aR,11aR)-9,10-dimethoxy-6a,11a-dihydro-6H-benzofurano[3,2-c] chromen-3-ol	64.26	0.55	0.42	HQ
MOL000387	HQ7	Bifendate	31.1	-0.06	0.67	HQ
MOL000392	HQ8	Formononetin	69.67	0.02	0.21	HQ
MOL000442	HQ9	1,7-Dihydroxy-3,9-dimethoxy pterocarpene	39.05	-0.04	0.48	HQ
MOL000358	B1	Beta-sitosterol	36.91	0.99	0.75	DG, GT, DZ, BS, HQIN, HB

MOL000449	B2	Stigmasterol	43.83	1	0.76	DG, DZ, HQIN, HB
MOL000359	C1	Sitosterol	36.91	0.87	0.75	GT, BS, HQIN
MOL008456	GT1	(3E,4R)-4-(1,3-benzodioxol-5-ylmethyl)-3-[(3,4,5-trimethoxyphenyl) methylidene] oxolan-2-one	51.78	-0.09	0.65	GT
MOL008457	GT2	Tetrahydroalstonine	32.42	0.33	0.81	GT
MOL008458	GT3	Angustidine	51.85	0.16	0.66	GT
MOL008460	GT4	Geissoschizinc acid	49.92	0.12	0.6	GT
MOL008463	GT5	SMR000232338	56.74	-0.19	0.75	GT
MOL008465	GT6	(E)-16,17-Didehydro-17-methoxy-17,18-seco-3-beta-yohimban-16-carboxylic acid methyl ester	32.75	0.82	0.64	GT
MOL008468	GT7	Methyl (E)-2-[(2S,3Z,12bS)-3-ethylidene-2,4,6,7,12,12b-hexahydro-1H-indolo[3,2-h] quinolizin-2-yl]-3-methoxyprop-2-enoate	56.83	0.37	0.64	GT
MOL008467	GT8	Rhynchophylline A	68.68	-0.15	0.69	GT
MOL008469	C2	Rhynchophylline	41.82	0.38	0.57	GT, SWT
MOL008470	GT9	SMR000232333	78.38	-0.24	0.75	GT
MOL008471	GT10	Isorhyncophylline	47.31	0.33	0.57	GT
MOL008472	GT11	Hirsutaside A	70.34	-0.1	0.81	GT
MOL008473	GT12	(E)-2-[(3S,6'S,7'S,8'aS)-6'-ethyl-2-keto-spiro[indoline-3,1'-indolizidine]-7'-yl]-3-methoxy-acrylic acid methyl ester	57.85	0.33	0.57	GT
MOL008474	GT13	(E)-2-[(3R,6'S,7'S,8'aS)-6'-ethyl-2-keto-spiro[indoline-3,1'-indolizidine]-7'-yl]-3-methoxy-acrylic acid methyl ester	54.47	0.25	0.57	GT
MOL008475	GT14	Mitraphyllic acid	31.7	-0.17	0.7	GT
MOL008476	GT15	Hirsutaside B	40.21	0.08	0.8	GT
MOL008477	GT16	Corynoxeine	57.13	0.5	0.57	GT
MOL008478	GT17	Methyl (E)-2-[(2S,3R,12bS)-3-vinyl-1,2,3,4,6,7,12,12b-octahydroindolo[3,2-h] quinolizin-2-yl]-3-methoxy-prop-2-enoate	31.94	0.61	0.64	GT
MOL008482	GT18	(2S,12bR)-methyl 2-((E)-1-oxobut-2-en-2-yl)-1,2,6,7,12,12b-hexahydroindolo[2,3-a] quinolizine-3-carboxylate	42.07	0.63	0.6	GT

MOL008484	GT19	Vincoside lactam_qt	50.81	-0.16	0.82	GT
MOL008487	C3	Hirsutine	34.44	0.78	0.43	GT, SWT
MOL008488	GT20	Yohimbine	46.42	-0.02	0.81	GT
MOL008489	GT21	Delta (sup 18)-Hirsutine	41.64	0.76	0.64	GT
MOL008490	GT22	Isocorynantheic acid	72.36	0.26	0.6	GT
MOL008635	GT23	Coryincine	38.27	-0.13	0.81	GT
MOL012921	DZ1	Stepharine	31.55	0.17	0.33	DZ
MOL012992	DZ2	Mauritine D	89.13	0.62	0.45	DZ
MOL001454	E1	Berberine	36.86	0.57	0.78	DZ, HB
MOL001522	DZ3	(S)-Coclaurine	42.35	0.06	0.24	DZ
MOL000627	DZ4	Stepholidine	33.11	0.29	0.54	DZ
MOL007213	DZ5	Nuciferin	34.43	0.83	0.4	DZ
MOL000787	E2	Fumarine	59.26	-0.13	0.83	DZ, HB
MOL002773	DZ6	Beta-carotene	37.18	1.52	0.58	DZ
MOL004624	WWZ1	Longikaurin A	47.72	0.09	0.53	WWZ
MOL005317	WWZ2	Deoxyharringtonine	39.27	-0.25	0.81	WWZ
MOL008956	WWZ3	Angeloylgomisin O	31.97	0.41	0.85	WWZ
MOL008957	WWZ4	Schizandrer B	30.71	0.15	0.83	WWZ
MOL008968	WWZ5	Gomisin-A	30.69	-0.02	0.78	WWZ
MOL008978	WWZ6	Gomisin R	34.84	0.04	0.86	WWZ
MOL008992	WWZ7	Wuweizisu C	46.27	0.5	0.84	WWZ
MOL001689	HQIN1	Acacetin	34.97	-0.05	0.24	HQIN
MOL000173	HQIN2	Wogonin	30.68	0.04	0.23	HQIN
MOL000228	HQIN3	(2R)-7-hydroxy-5-methoxy-2-phenylchroman-4-one	55.23	0.26	0.2	HQIN
MOL002714	HQIN4	Baicalein	33.52	-0.05	0.21	HQIN
MOL002913	HQIN5	Dihydrobaicalin_qt	40.04	0.18	0.21	HQIN
MOL002915	HQIN6	Salvigenin	49.07	-0.03	0.33	HQIN
MOL002917	HQIN7	5,2',6'-Trihydroxy-7,8-dimethoxyflavone	45.05	-0.11	0.33	HQIN
MOL002927	HQIN8	Skullcapflavone II	69.51	-0.07	0.44	HQIN
MOL002928	HQIN9	Oroxylin a	41.37	0.13	0.23	HQIN
MOL002932	HQIN10	Panicolin	76.26	0.31	0.29	HQIN
MOL002934	HQIN11	Neobaicalein	104.34	-0.19	0.44	HQIN

MOL002937	HQIN12	Dihydrooroxylin	66.06	0.13	0.23	HQIN
MOL000525	HQIN13	Norwogonin	39.4	-0.17	0.21	HQIN
MOL000552	HQIN14	5,2'-Dihydroxy-6,7,8-trimethoxyflavone	31.71	0	0.35	HQIN
MOL001458	D2	Coptisine	30.67	0.32	0.86	HQIN, HB
MOL001490	HQIN15	Bis[(2S)-2-ethylhexyl] benzene-1,2-dicarboxylate	43.59	0.68	0.35	HQIN
MOL002879	HQIN16	Diop	43.59	0.26	0.39	HQIN
MOL002897	D1	Epiberberine	43.09	0.4	0.78	HQIN, FXM
MOL008206	HQIN17	Moslosooflavone	44.09	0.54	0.25	HQIN
MOL010415	HQIN18	11,13-Eicosadienoic acid, methyl ester	39.28	1.24	0.23	HQIN
MOL012266	HQIN19	Rivularin	37.94	-0.13	0.37	HQIN
MOL002643	HB1	Delta 7-stigmastenol	37.42	0.83	0.75	HB
MOL002644	HB2	Phellopterin	40.19	0.48	0.28	HB
MOL002651	HB3	Dehydrotanshinone II A	43.76	0.52	0.4	HB
MOL002662	HB4	Rutaecarpine	40.3	0.71	0.6	HB
MOL002663	HB5	Skimmianin	40.14	1.1	0.2	HB
MOL002666	HB6	Chelerythrine	34.18	0.28	0.78	HB
MOL002668	HB7	Worenine	45.83	0.24	0.87	HB
MOL002670	HB8	Cavidine	35.64	0.63	0.81	HB
MOL000622	HB9	Magnograndiolide	63.71	-0.24	0.19	HB
MOL000785	E3	Palmatine	64.6	0.37	0.65	HB, SWT
MOL000790	HB10	Isocorypalmine	35.77	0.43	0.59	HB
MOL001455	HB11	(S)-Canadine	53.83	0.64	0.77	HB
MOL001771	HB12	Poriferast-5-en-3beta-ol	36.91	1.14	0.75	HB
MOL002894	HB13	Berberrubine	35.74	0.17	0.73	HB
MOL005438	HB14	Campesterol	37.58	0.95	0.71	HB
MOL006422	HB15	Thalifendine	44.41	0.21	0.73	HB
434-22-0	DLG1	Nandrolone	High	YES	YES	DLG
31147-56-5	DLG2	D-campholic acid	High	YES	YES	DLG
10481-92-2	DLG3	Amurensine	High	YES	YES	DLG
124-76-5	DLG4	Isoborneol	High	YES	YES	DLG
4335-12-0	DLG5	Toddaculin	High	YES	YES	DLG

31524-62-6	DLG6	Isobavachin	High	YES	YES	DLG
523-50-2	DLG7	Angelicin	High	YES	YES	DLG
100858-26-2	DLG8	(1R,3aR,7aR)-1-((2R, 5S, Z)-6-hydroxy-5,6-dimethylhept-3-en-2-yl)-7a-methyloctahydro-4H-inden-4-one	High	YES	YES	DLG
41060-15-5	DLG9	Neobavaisoflavone	High	YES	YES	DLG
56083-03-5	DLG10	Isobavachromene	High	YES	YES	DLG
95716-67-9	DLG11	(1R,3aR,4S,7aR)-1-[(2R,3E,5S)-6-Hydroxy-5,6-dimethyl-3-hepten-2-yl]-7a-methyloctahydro-1H-inden-4-ol	High	YES	YES	DLG
163217-09-2	DLG12	Inecalcitol	High	YES	YES	DLG
103909-75-7	DLG13	Maxacalcitol	High	YES	YES	DLG
19879-32-4	DLG14	Bavachin	High	YES	YES	DLG
19879-30-2	DLG15	Bavachinin A	High	YES	YES	DLG
41743-38-8	DLG16	Bavachromene	High	YES	YES	DLG
170900-13-7	DLG17	Paratocarpin K	High	YES	YES	DLG
66-97-7	DLG18	Psoralen	High	YES	YES	DLG
10309-37-2	DLG19	Bakuchiol	High	YES	YES	DLG
10161-33-8	DLG20	Trenbolone	High	YES	YES	DLG
3764-87-2	DLG21	Trestolone	High	YES	YES	DLG
464-45-9	DLG22	(-)-Borneo	High	YES	YES	DLG
3704/9/4	DLG23	Mibolerone	High	YES	YES	DLG
434-07-1	DLG24	Oxymetholone	High	YES	YES	DLG
10418-03-8	DLG25	Stanozolol	High	YES	YES	DLG
5630-53-5	DLG26	Tibolone	High	YES	YES	DLG
517-66-8	DML1	Dicentrine	High	YES	YES	DML
28832-07-7	DML2	(+)-Dicentrine	High	YES	YES	DML
485-49-4	DML3	(+)-Bicuculline	High	YES	YES	DML
607-80-7	DML4	Sesamin	High	YES	YES	DML
61263-49-8	DML5	Vitexilactone	High	YES	YES	DML
83924-98-5	ZZM1	Flavidin	High	YES	YES	ZZM
61825-98-7	ZZM2	Sipeimine	High	YES	YES	ZZM
Peimisine	ZZM3	Peimisine	High	YES	YES	ZZM
18059-10-4	ZZM4	Peiminine	High	YES	YES	ZZM

23496-41-5	ZZM5	Peimine	High	YES	YES	ZZM
10351-88-9	ZZM6	phyllanthin	High	YES	YES	ZZM
10178-31-1	ZZM7	trans-Communol	High	YES	YES	ZZM
98774-23-3	ZZM8	Tesmilifene	High	YES	YES	ZZM
98243-57-3	ZZM9	Hupehenine	High	YES	YES	ZZM
308081-08-5	CS1	Atipamezole	High	YES	YES	CS
72510-04-4	CS2	Ungeremine	High	YES	YES	CS
1338-24-5	CS3	Naphthenic acids	High	YES	YES	CS
59870-68-7	ZGC1	Glabridin	High	YES	YES	ZGC
961-29-5	ZGC2	Isoliquiritigenin	High	YES	YES	ZGC
41983-91-9	ZGC3	Glabranin	High	YES	YES	ZGC
34221-41-5	ZGC4	Retrochalcone	High	YES	YES	ZGC
142561-10-2	ZGC5	Glyasperin D	High	YES	YES	ZGC
63529-06-6	ZGC6	4,2'-Dihydroxy-4'-methoxychalcone	High	YES	YES	ZGC
51828-10-5	ZGC7	2'-O-Methylisoliquiritigenin	High	YES	YES	ZGC
151135-64-7	ZGC8	4'-Hydroxy-2,4-dimethoxychalcone	High	YES	YES	ZGC
58749-22-7	ZGC9	Licochalcone A	High	YES	YES	ZGC
578-86-9	ZGC10	Liquiritigenin	High	YES	YES	ZGC
5489-57-6	FXM1	Arborinine	High	YES	YES	FXM
190595-66-5	FXM2	(3'S,3R,4S)-Desfluoro Ezetimibe	High	YES	YES	FXM
64795-23-9	FXM3	Etisulergine	High	YES	YES	FXM
1700622-07-6	FXM4	(3R,4S)-1-(4-fluorophenyl)-3-((S)-3-hydroxy-3-(p-tolyl) propyl)-4-(4-hydroxyphenyl) azetidin-2-one	High	YES	YES	FXM
163380-16-3	FXM5	(R)-Ezetimibe	High	YES	YES	FXM
74805-91-7	FXM6	Methylophiopogonanone B	High	YES	YES	FXM
1700622-06-5	FXM7	Ezetimibe 3-Fluoro Impurity	High	YES	YES	FXM
1700622-06-5	FXM8	(3S,4S,3'R)-Ezetimbe	High	YES	YES	FXM
1700622-08-7	FXM9	(3R,4S)-1-(4-chlorophenyl)-3-((S)-3-(4-fluorophenyl)-3-hydroxypropyl)-4-(4-hydroxyphenyl) azetidin-2-one	High	YES	YES	FXM
1593543-00-0	FXM10	(3'S)-ent-Ezetimibe	High	YES	YES	FXM
1093659-90-5	FXM11	Ezetimibe D4	High	YES	YES	FXM

191330-56-0	FXM12	EzetiMibe Ketone	High	YES	YES	FXM
361-37-5	FXM13	Methysergide	High	YES	YES	FXM
28787-36-2	FXM14	Maltol acetate	High	YES	YES	FXM
1798008-25-9	FXM15	Ezetimibe Fluoro Isomer	High	YES	YES	FXM
81409-90-7	FXM16	Cabergoline	High	YES	YES	FXM
26171-23-3	FXM17	1-Methyl-5-p-toluoylpyrrole-2-acetic acid	High	YES	YES	FXM
113-42-8	FXM18	Methylergometrine	High	YES	YES	FXM
37686-84-3	FXM19	Trans-Dihydro Lisuride	High	YES	YES	FXM
83455-48-5	FXM20	Bromerguride	High	YES	YES	FXM
83455-48-5	FXM21	Metergoline	High	YES	YES	FXM
539-15-1	FXM22	Hordenine	High	YES	YES	FXM
77650-95-4	FXM23	Proterguride	High	YES	YES	FXM
59091-65-5	FXM24	Delergotrile	High	YES	YES	FXM
121588-75-8	FXM25	Amesergide	High	YES	YES	FXM
957-66-4	SWT1	Isolinderalactone	High	YES	YES	SWT
139122-81-9	SWT2	Tripterifordin	High	YES	YES	SWT
18556-27-9	SWT3	Cyclanoline	High	YES	YES	SWT
5890-28-8	SWT4	Cassythicine	High	YES	YES	SWT
517-63-5	SWT5	Stephanine	High	YES	YES	SWT
6879-02-3	SWT6	Tylocrebrine	High	YES	YES	SWT
4030-51-7	SWT7	Cassyfiline	High	YES	YES	SWT
83-79-4	SWT8	Rotenone	High	YES	YES	SWT
142937-50-6	SWT9	Triptoquinone B	High	YES	YES	SWT

Mol: Molecular; OB: Oral bioavailability; GIA: GI absorption; BBB: Blood-brain barrier permeability; DL: Drug-likeness.

Supplementary Table 3 Potential signaling pathways of LMQXM for ADHD (Log P＜-5.0).

Term	Pathway	Log P	Count	Target
hsa04726	Serotonergic synapse	-14.81	10	APP, CYP2D6, HTR1A, HTR2A, HTR2C, HTR3A, MAOA, MAOB, SLC6A4, SLC18A2

hsa04080	Neuroactive ligand-receptor interaction	-14.36	13	ADRB2, CHRM1, CHRNA4, CHRNA7, CNR1, DRD1, DRD2, GRIA2, NR3C1, HTR1A, HTR2A, HTR2C, OPRM1
hsa04151	PI3K-Akt signaling pathway	-14.34	13	AKT1, CHRM1, FGFR1, MTOR, IGF2, IL6, JAK2, MET, MYC, NGF, NOS3, PIK3CA, TP53
hsa04020	Calcium signaling pathway	-13.18	11	ADRB2, CHRM1, CHRNA7, DRD1, FGFR1, HTR2A, HTR2C, MET, NGF, NOS3, CXCR4
hsa04728	Dopaminergic synapse	-12.37	9	AKT1, DRD1, DRD2, FOS, GRIA2, MAOA, MAOB, SLC6A3, SLC18A2
hsa04725	Cholinergic synapse	-11.16	8	ACHE, AKT1, CHRM1, CHRNA4, CHRNA7, FOS, JAK2, PIK3CA
hsa04024	cAMP signaling pathway	-10.35	9	ADRB2, AKT1, CHRM1, DRD1, DRD2, FOS, GRIA2, HTR1A, PIK3CA
hsa05163	Human cytomegalovirus infection	-10.28	9	AKT1, CTNNB1, MTOR, IL6, MYC, PIK3CA, TNF, TP53, CXCR4
hsa05010	Alzheimer disease	-9.58	10	AKT1, APP, CHRM1, CHRNA7, CTNNB1, MTOR, IL6, PIK3CA, SNCA, TNF
hsa04010	MAPK signaling pathway	-9.25	9	AKT1, FGFR1, FOS, IGF2, MET, MYC, NGF, TNF, TP53
hsa04919	Thyroid hormone signaling pathway	-9.19	7	AKT1, CTNNB1, ESR1, MTOR, MYC, PIK3CA, TP53
hsa04015	Rap1 signaling pathway	-9.00	8	AKT1, CNR1, CTNNB1, DRD2, FGFR1, MET, NGF, PIK3CA
hsa05022	Pathways of neurodegeneration - multiple diseases	-8.68	10	APP, CHRM1, CHRNA7, CTNNB1, MTOR, GRIA2, IL6, SLC6A3, SNCA, TNF
hsa01521	EGFR tyrosine kinase inhibitor resistance	-8.64	6	AKT1, MTOR, IL6, JAK2, MET, PIK3CA
hsa05012	Parkinson disease	-8.20	8	DRD1, DRD2, MAOA, MAOB, SLC6A3, SLC18A2, SNCA, TP53
hsa04668	TNF signaling pathway	-7.73	6	AKT1, FOS, IL6, MMP9, PIK3CA, TNF

hsa04210	Apoptosis	-7.22	6	AKT1, FOS, NGF, PIK3CA, TNF, TP53
hsa04550	Signaling pathways regulating pluripotency of stem cells	-7.09	6	AKT1, CTNNB1, FGFR1, JAK2, MYC, PIK3CA
hsa04630	JAK-STAT signaling pathway	-6.77	6	AKT1, MTOR, IL6, JAK2, MYC, PIK3CA
hsa04540	Gap junction	-6.63	5	DRD1, DRD2, GJA1, HTR2A, HTR2C
hsa04620	Toll-like receptor signaling pathway	-6.27	5	AKT1, FOS, IL6, PIK3CA, TNF
hsa05169	Epstein-Barr virus infection	-6.21	6	AKT1, IL6, MYC, PIK3CA, TNF, TP53
hsa04066	HIF-1 signaling pathway	-6.17	5	AKT1, MTOR, IL6, NOS3, PIK3CA
hsa04935	Growth hormone synthesis, secretion, and action	-5.98	5	AKT1, FOS, MTOR, JAK2, PIK3CA
hsa04071	Sphingolipid signaling pathway	-5.98	5	AKT1, NOS3, PIK3CA, TNF, TP53
hsa04014	Ras signaling pathway	-5.86	6	AKT1, FGFR1, IGF2, MET, NGF, PIK3CA
hsa00982	Drug metabolism - cytochrome P450	-5.35	4	CYP2D6, CYP3A4, MAOA, MAOB
hsa04721	Synaptic vesicle cycle	-5.21	4	SLC6A2, SLC6A3, SLC6A4, SLC18A2
hsa05164	Influenza A	-5.21	5	AKT1, IL6, JAK2, PIK3CA, TNF
hsa05168	Herpes simplex virus 1 infection	-5.03	7	AKT1, MTOR, IL6, JAK2, PIK3CA, TNF, TP53

2 Supplementary Figures

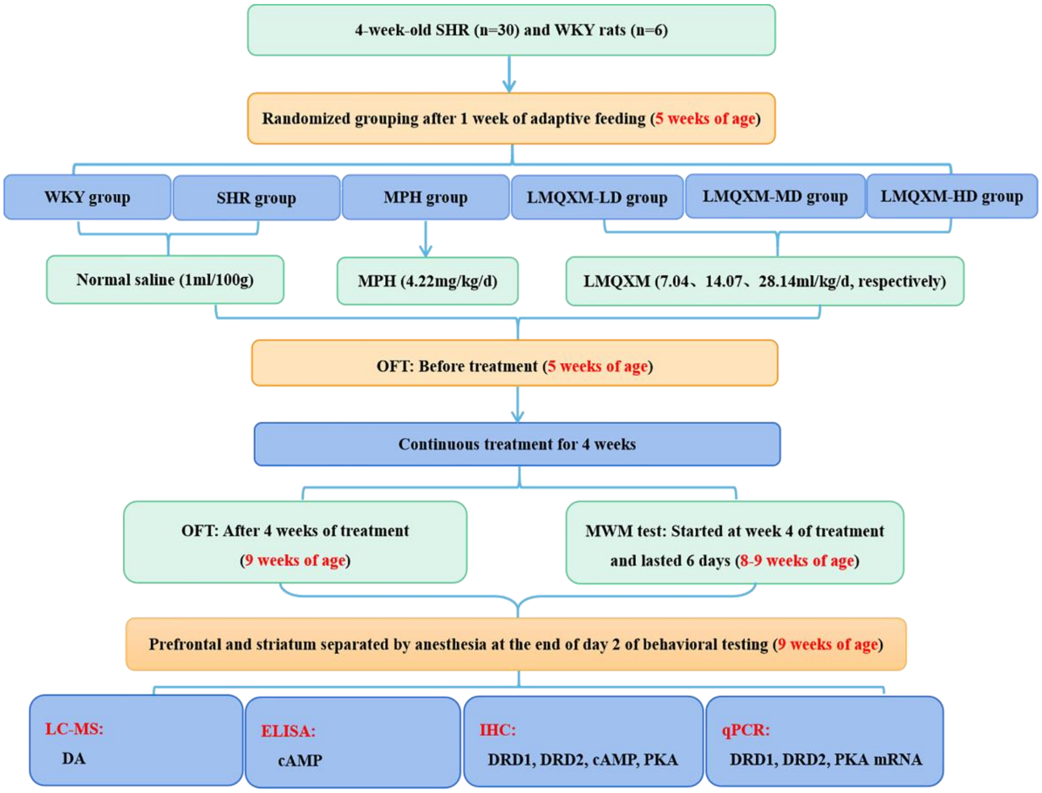

Supplementary Figure 1. The flow chart of the animal experimental study. We performed OFT at 5 and 9 weeks of age, MWM test at 8 weeks of age, LC-MC, ELISA, IHC, and qPCR at 9 weeks of age in rats.

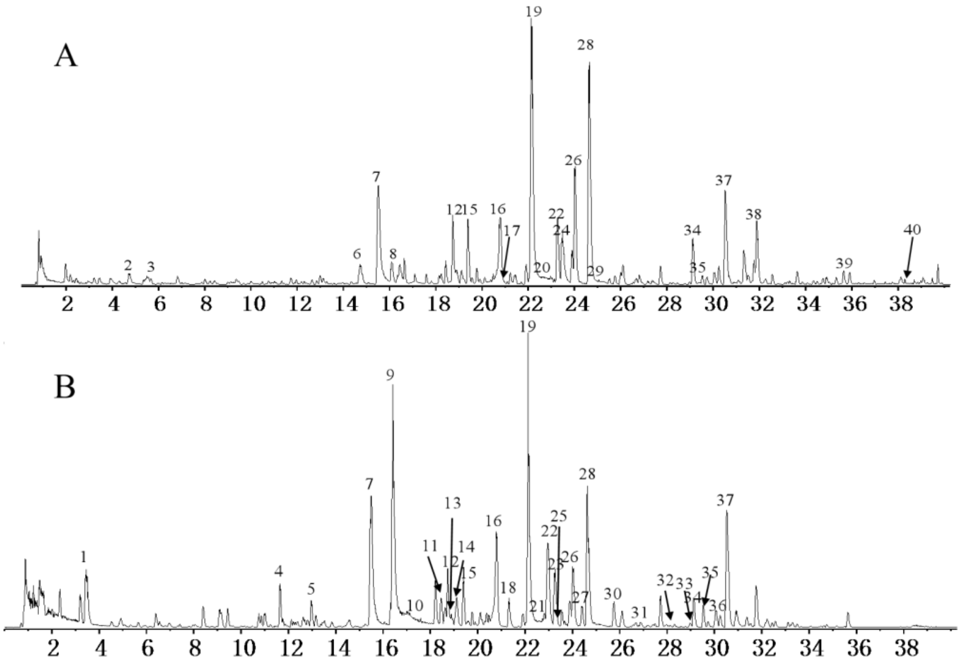

Supplementary Figure 2. Base peak ion flow diagram of LMQXM: The representative numbers of each compound in the figure correspond to the numbering in **Supplementary Table 1.** (A) Positive ion mode. (B) Negative ion mode.
